# CKMT1 deficiency contributes to mitochondrial dysfunction and promotes intestinal epithelial cell apoptosis via reverse electron transfer-derived ROS in colitis

**DOI:** 10.1038/s41419-025-07504-4

**Published:** 2025-03-15

**Authors:** Zhijie Wang, Haicong Wu, Xin Chang, Yihang Song, Yan Chen, Ziwei Yan, Lun Gu, Ruxi Pang, Tian Xia, Zixuan He, Zhaoshen Li, Shuling Wang, Yu Bai

**Affiliations:** 1 National Clinical Research Center for Digestive Diseases, Department of Gastroenterology, Changhai Hospital, Naval Medical University, Shanghai, China; 2 Department of Gastroenterology, Affiliated Hangzhou First People’s Hospital, Westlake University School of Medicine, Hangzhou, China; 3 National Key Laboratory of Immunology and Inflammation, Naval Medical University, Shanghai, China; 4 Changhai Clinical Research Unit, Changhai Hospital, Naval Medical University, Shanghai, China; 5 Department of Gastroenterology, The Seventh Affiliated Hospital of Southern Medical University, Foshan, China; 6 Institute of Immunology and the CAS Key Laboratory of Innate Immunity and Chronic Disease, Division of Life Sciences and Medicine, University of Science and Technology of China, Hefei, China

**Keywords:** Apoptosis, Ulcerative colitis

## Abstract

Mitochondrial dysfunction contributes to the pathogenesis of ulcerative colitis (UC). As a mitochondrial isozyme of creatine kinases, which control energy metabolism, CKMT1 is thought to be a critical molecule in biological processes. However, the specific role of CKMT1 in intestinal inflammation remains largely unknown. Here, we observed markedly decreased CKMT1 expression in the colon tissues of UC patients and dextran sodium sulfate (DSS)-induced colitis mice. We generated intestinal epithelial-specific CKMT1 knockout mice and demonstrated the key role of CKMT1 in mitochondrial homeostasis, intestinal epithelial barrier function, oxidative stress, and apoptosis. In the in vitro experiments, CKMT1 expression limited the activation of the intrinsic and extrinsic apoptotic pathways in IECs. Mechanistically, the loss of CKMT1 expression in IECs increased TNF-α-induced mitochondrial reactive oxygen species (ROS) generation via reverse electron transfer (RET). RET-ROS promoted mitochondrial permeability transition pore (mPTP) opening, ultimately resulting in cell apoptosis during intestinal inflammation. In conclusion, our data demonstrated that CKMT1 is important in maintaining intestinal homeostasis and mitochondrial function. This study provides a promising basis for future research and a potential therapeutic target for inflammatory bowel disease (IBD).

## Introduction

Mitochondria are increasingly considered master regulators of inflammation [1]. Numerous studies have shown that mitochondrial dysfunction is highly involved in various complex chronic inflammatory processes, such as acute pancreatitis and Parkinson’s disease [2–4]. Mitochondrial function also plays a key role in inflammatory bowel disease (IBD) [5], a disorder characterized by chronic gastrointestinal tract inflammation, primarily Crohn’s disease (CD) and ulcerative colitis (UC). Previous studies have revealed that approximately 5% (29/574) of the genome-wide significant IBD locus (within 100 kb) identified by genome-wide association studies is strongly associated with mitochondrial function [6, 7]. Recent studies have also demonstrated that knocking out several genes encoding crucial mitochondrial proteins, such as voltage-dependent anion channel 1 (VDAC1) [8] and prohibitin 1 (PHB1) [9], can disrupt mitochondrial function and aggravate experimental colitis in mice. Moreover, the severity of mitochondrial structure destruction positively correlates with the severity of intestinal inflammation in experimental colitis mice [10]. Abnormal mitochondrial morphology has been observed in inflamed or even noninflamed colonic tissue biopsies of UC and CD patients [10, 11], indicating that mitochondrial dysfunction occurs early in IBD development and acts as a forerunner of inflammation.

It is well-known that creatine kinases (CKs) can catalyze phosphate group transfer between ATP and phosphocreatine (PCr), playing a central role in temporal and spatial buffering and cellular energetics regulation [12], which is particularly important for cells with vigorous metabolism or high energy fluctuations, such as intestinal epithelial cells (IECs) and myocardial cells. Interestingly, intestinal biopsy transcriptional data revealed a dysregulated PCr/CK metabolic system among IBD patients [13]. Cytosolic CK isoforms (CKM and CKB) in IECs have been reported to significantly affect intestinal epithelial homeostasis and murine colitis mainly through mucosal barrier regulation mediated by the PCr/CK system [13]. Mitochondrial creatine kinase 1 (CKMT1) is the mitochondrial isozyme of CKs and highly expressed in the intestinal tract. However, evidence for the role of CKMT1 in IBD pathogenesis is largely lacking. Considering that different subcellular localizations endow protein isoforms with divergent functional properties, exploring the potential relationship between CKMT1 and IBD is of substantial interest.

In this study, the downregulated CKMT1 protein expression in colon of UC patients and dextran sodium sulfate (DSS)-induced colitis mice was reported. We next generated intestinal epithelial-specific *Ckmt1*-knockout mice, and found that CKMT1 deficiency aggravated DSS-induced colitis and impaired mitochondrial function. Mechanistically, our data demonstrated that CKMT1 expression could limit apoptotic pathway activation and the generation of mitochondrial reverse electron transport (RET)-derived reactive oxygen species (ROS) in IECs, suggesting that CKMT1 is a critical contributor to intestinal homeostasis.

## Results

### CKMT1 protein expression decreased in UC

**Fig. 1:**
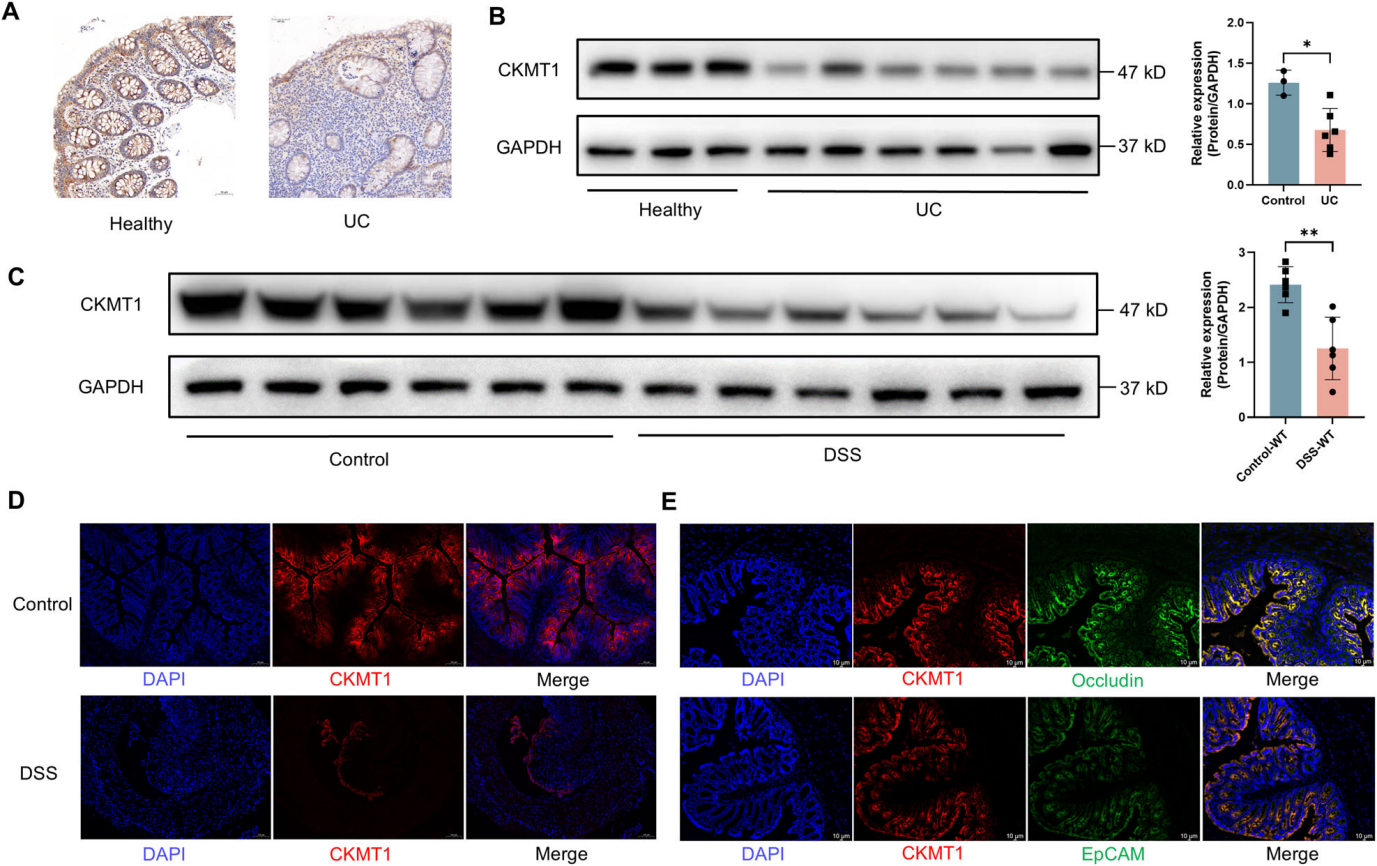
CKMT1 protein was down-regulated in UC and experimental colitis. **A** Representative images of immunohistochemical staining of colon sections from UC and healthy. **B** Western blotting analysis of CKMT1 in colonic biopsy tissues from UC (n = 6) and healthy (n = 3), and **C** in colonic tissues from DSS-induced colitis mice and controls (n = 6). **D** Representative images of immunofluorescence staining of CKMT1 in colon sections from DSS-induced colitis mice and controls. **E** Immunofluorescence staining analysis of the co-localization of CKMT1 and Occludin/EpCAM. *P < 0.05; **P < 0.01; ***P < 0.001.

First, we assessed the protein expression of CKMT1 across diverse human body tissues using the Human Protein Atlas project data [14]. CKMT1 was found to be highly expressed throughout the gastrointestinal tract, including the esophagus, small intestine, and colon (Fig. S1A), implying an indispensable role of CKMT1 plays in maintaining physiological functions in the gut. To explore the potential effect of CKMT1 on intestinal inflammation, we subsequently analyzed its expression in colon tissues of UC patients. Immunohistochemical results revealed that CKMT1 protein expression was clearly lower in the UC patients’ biopsy specimens than in those from healthy controls (Fig. 1A). Similar results were observed by Western blotting analysis (Fig. 1B). We then evaluated CKMT1 expression in a mouse model of DSS-induced UC. Compared to those in the control group, significant weight loss (Fig. S1B), increased disease activity index (DAI) scores (Fig. S1C), colon shortening (Fig. S1D), and histologic damage (Fig. S1E) were observed in the wild-type mice exposed to DSS. These results suggested the successful establishment of DSS-induced colitis in the mice. Colonic CKMT1 protein expression was demonstrated to be significantly downregulated during DSS-induced colitis in Western blotting analysis (Fig. 1C) and immunofluorescence (IF) staining (Fig. 1D and Fig S1F). In addition, the consistency with human UC indicated the applicability of using the DSS-induced acute colitis mouse model for CKMT1 research. To further explore the role of CKMT1 in intestinal inflammation, we performed IF staining for CKMT1 in the colon tissues of wild-type mice. The results revealed extensive co-localization between CKMT1 and Occludin or EpCAM (Fig. 1E), indicating that CKMT1 is mainly expressed in IECs.

These data collectively demonstrate that CKMT1 protein expression is downregulated in UC.

### Epithelial CKMT1 depletion causes no obvious phenotype

**Fig. 2:**
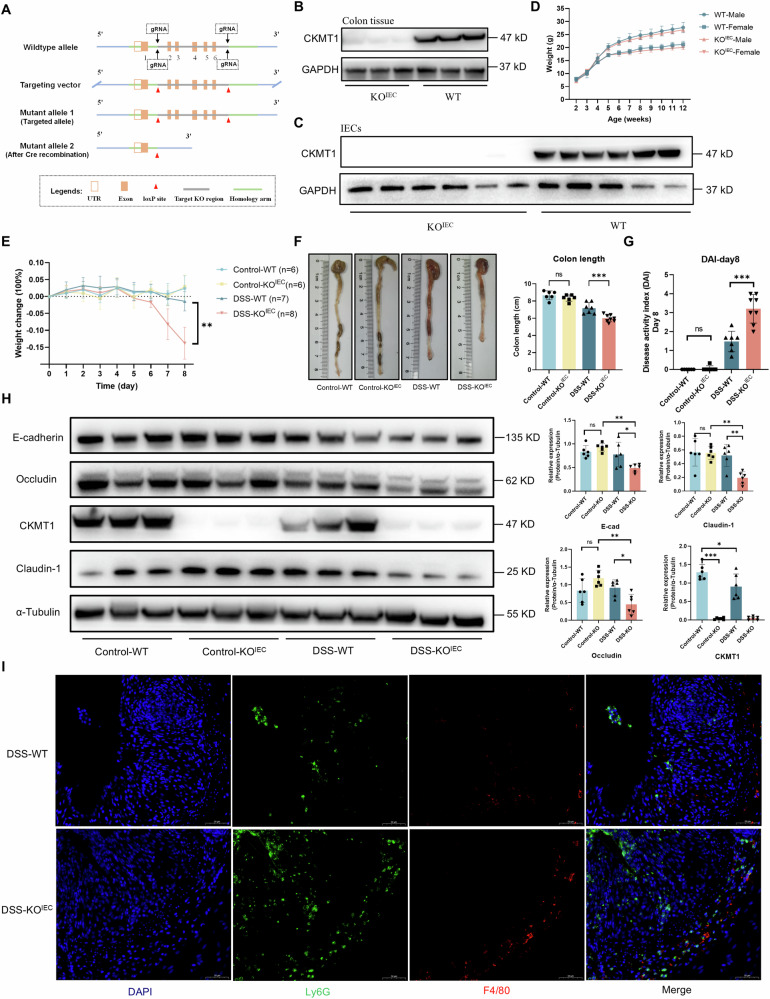
Epithelial CKMT1 deficiency aggravated DSS-induced colitis in mice. **A** Targeting strategy for mouse conditional *Ckmt1* knockout. (B-C) The knockout was verified by Western blotting in colon tissues (**B**) and isolated primary IECs (**C**) from mice. **D** Weight curves showing similar growth of male and female CKMT1 KO^IEC^ and WT mice (n = 5 for each group). **E** Weight loss following DSS treatment was aggravated after epithelial CKMT1 deletion (n = 6–8). **F** Representative megascopic viewing of colon, and colon length. **G** DAI scores at day 8. **H** Evaluation of intestinal barrier function (E-Cadherin, Occludin, and Claudin-1) via Western blotting (n = 6). (**I**) Immunofluorescent staining of Ly6G and F4/80 in the colon tissues. *P < 0.05; **P < 0.01; ***P < 0.001.

We next generated intestinal epithelial-specific *Ckmt1* knockout (*Ckmt1*^flox/flox, Vil-Cre^, hereafter referred to as KO^IEC^) mice by crossing *Ckmt1*-flox mice (Fig. 2A) with Vil1-Cre mice, and the knockout was verified by genotyping (data not shown) and Western blotting (Fig. 2B, C). Unexpectedly, CKMT1 deficiency appeared to have no effect on mouse growth, as KO^IEC^ mice gained weight nearly equally compared to littermate controls (*Ckmt1*^flox/flox^, hereafter referred to as WT) (Fig. 2D). Furthermore, mucosal architectures of the small and large intestines were indistinguishable between KO^IEC^ and WT mice (Fig. S2A). We also investigated whether CKMT1 deficiency affected intestinal barrier function; however, no significant differences were detected between the genotypes, as indicated by Alcian blue and periodic acid-Schiff (AB/PAS) staining of the colon tissues (Fig. S3B). We also did not find any other obvious phenotypes or spontaneous diseases in CKMT1 KO^IEC^ mice. Finally, we extracted intestinal crypts from KO^IEC^ and WT mice to generate intestinal organoids. The morphology and growth of the organoids were indistinguishable between the two groups (Fig. S2B).

### CKMT1 deficiency renders mice more susceptible to DSS-induced colitis

Previous studies have demonstrated that the phenotypes of mice with ablation of key gene, such as *Tjp1* and *Ocln*, are not always easily identifiable, unless the mice are exposed to various stresses [15, 16]. Thus, we treated CKMT1 KO^IEC^ and WT mice with DSS. As expected, a low concentration of DSS (2%) was sufficient to cause dramatic weight loss in KO^IEC^ mice, while it only slightly affected WT mice (Fig. 2E). CKMT1 KO^IEC^ mice exhibited more severe colitis than did WT mice, as evidenced by shorter colon length (Fig. 2F), higher DAI scores (Fig. 2G), and more severe histopathological damage to the colon (Fig. S3A). Additionally, we evaluated intestinal barrier function by Western blotting. Following DSS treatment, expression levels of E-cadherin, Occludin, and Claudin-1 were significantly lower in the colon tissues of KO^IEC^ mice than in those of WT mice (Fig. 2H). Similarly, AB/PAS staining of intracellular mucin in the colon suggested significantly compromised barrier function in KO^IEC^ mice (Fig. S3B). We observed increased levels of pro-inflammatory factors (IL-1β, TNF-α, and IL-17) in the colon tissues of KO^IEC^ mice compared to those in WT mice during DSS-induced colitis, via the enzyme-linked immunosorbent assay (ELISA) (Fig. S3C) and quantitative real-time PCR (qPCR) (Fig. S3D). Compared to DSS-treated WT mice, the number of colonic infiltrating neutrophils (Ly6G + ) and macrophages (F4/80 + ) were substantially increased in DSS-treated KO^IEC^ mice (Figs. 2I and S3E), and the protein levels of neutrophil elastase (NE) and myeloperoxidase (MPO) in colon tissues of KO^IEC^ mice were dramatically increased (Fig. S3F). At last, pro-inflammatory M1 macrophages (CD86 + ) were also obviously increased in the colon of KO^IEC^ mice (Fig. S3G, H). These data reflected a significantly increased colonic inflammation in KO^IEC^ mice.

Collectively, the above results demonstrate that epithelial CKMT1 deficiency aggravates DSS-induced colitis in mice.

### CKMT1 deficiency results in mitochondrial dysfunction during colitis

A great deal of evidence implicates that mitochondrial dysfunction is an important cause of IBD [5]. We firstly assessed the colonic expression of proteins related to mitochondrial homeostasis (or mitochondrial quality control), which are fundamental for maintaining mitochondrial function, in DSS-induced WT mice. Notably, the expression of PGC-1α (encoded by *Ppargc1a*), the master regulator of mitochondrial biogenesis, was significantly decreased after DSS treatment (Fig. S4A). The expression levels of Fis1, which is involved in fission, and Mfn1, which is involved in fusion, were significantly increased after DSS treatment (Fig. S4A). Besides, we assessed the activity of the mitochondrial antioxidant system, a key regulatory mechanism of mitochondrial homeostasis at the molecular level. The expressions of Prdx5, Gpx1, Txnrd2, and Prdx3 were significantly increased after DSS treatment, while no obvious changes were found regarding Sod2 and Gpx4 (Fig. S4B). These data provided further corroboration about the key role of mitochondrial dysfunction in IBD.

**Fig. 3:**
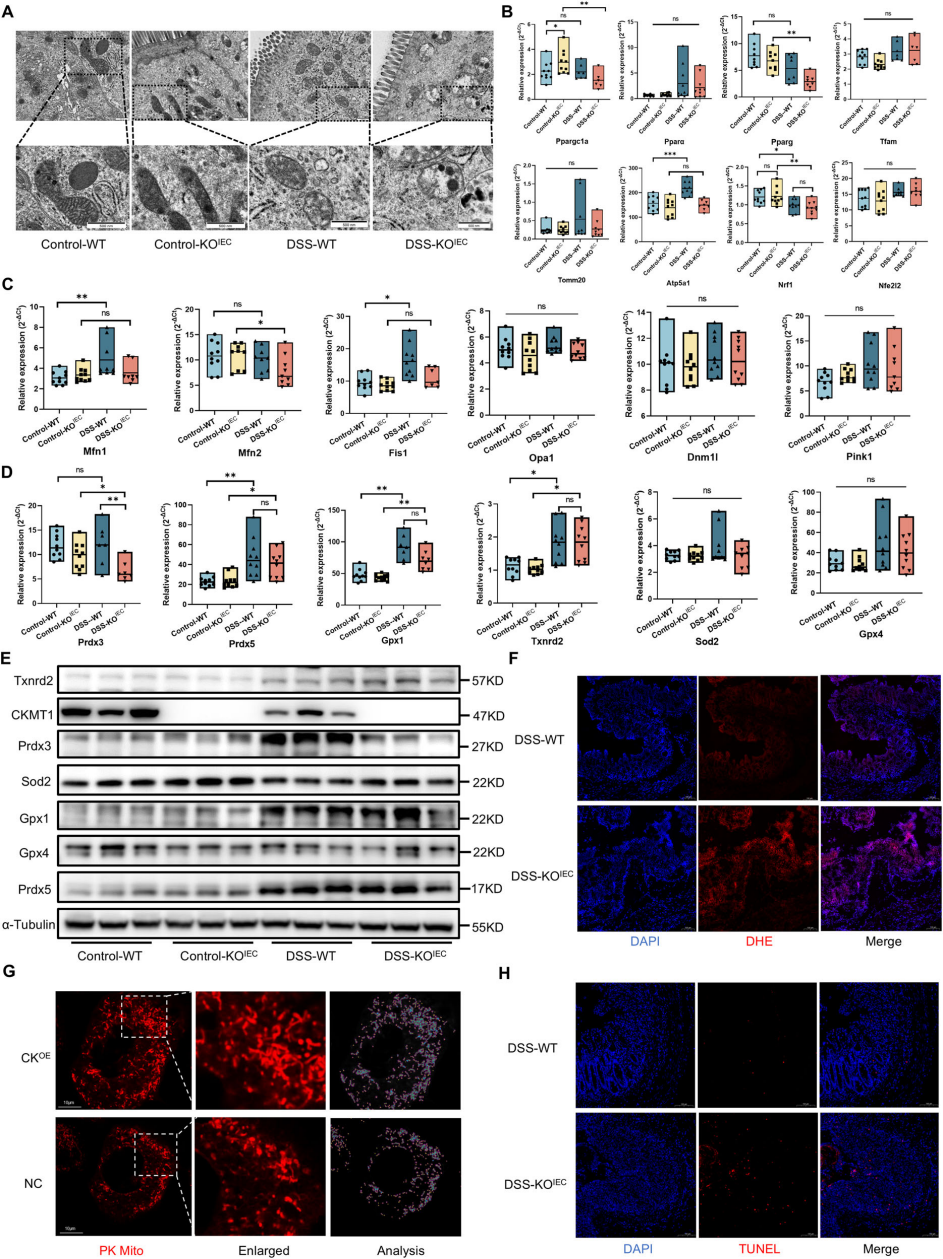
Epithelial CKMT1 deficiency aggravated mitochondrial dysfunction and apoptosis in colitis. **A** Representative images by transmission electron microscopy showing mitochondrial structural changes in colonic IECs of mice. Quantitative real-time PCR analysis of the expression of key genes involved in **B** mitochondrial biogenesis (n = 6–10), **C** mitochondrial dynamics and mitophagy (*Pink1*) (n = 7–10), and **D** mitochondrial antioxidant system (n = 7–10). **E** Western blotting analysis for the protein expressions of mitochondrial antioxidant genes in colon tissues. **F** Dihydroethidium (DHE) staining showing ROS levels in colon tissues. **G** Mitochondrial morphology of NCM460 cells with CKMT1 overexpression (CK^OE^) or control (NC) after TNF-α (50 ng/ml) and CHX (20 μg/mL) treatment for 1 h, was observed via confocal microscopy and analyzed via Image-J software. **H** TUNEL staining showing the apoptosis levels in colon tissues. *P < 0.05; **P < 0.01; ***P < 0.001. One-way ANOVA with a post hoc LSD test was used for pairwise comparisons.

Considering the critical role of CKMT1 in mitochondrial energy metabolism [17, 18], we presumed that CKMT1 deletion would exacerbate mitochondrial dysfunction during DSS-induced colitis. Thus, we first examined the ultrastructure of mitochondria in mouse IECs via transmission electron microscopy. In the control groups not subjected to DSS, we did not observe obvious abnormalities in the size or shape of the mitochondria between the WT and KO^IEC^ mice; however, a slight loss of clearly defined cristae structures seemed to be present in the KO^IEC^ mice (Fig. 3A). DSS treatment aggravated mitochondrial injury in KO^IEC^ mice compared to that in WT mice, characterized by mitochondrial swelling, a mottled matrix, and membrane rupture (Fig. 3A). We next evaluated the effect of CKMT1 deficiency on mitochondrial homeostasis via qPCR. Colon tissues were collected to analyze the mRNA levels of genes related to the regulatory mechanisms of mitochondrial homeostasis at the organelle level, including genes related to mitochondrial biogenesis, mitochondrial dynamics, and mitophagy [19, 20]. Notably, the expression of *Ppargc1a* and *Pparg* were significantly decreased in the KO^IEC^ mice after DSS treatment; however, no significant changes occurred in the WT mice (Fig. 3B). In addition, the expression of mitochondrial marker *Atp5a1* in WT mice was significantly upregulated after DSS exposure, while it failed to elevate in KO^IEC^ mice (Fig. 3B). CKMT1 knockout did not significantly impact the expressions of *Nrf1*, *Ppara*, *Tfam*, *Tomm20*, or *Nfe2l2* during colitis (Fig. 3B). These data indicated impaired mitochondrial biogenesis after CKMT1 knockout. We then turned to investigated mitochondrial dynamics. In WT mice, the expression levels of *Fis1* and *Mfn1* were significantly increased after DSS treatment, likely representing a protective mechanism in response to injury (Fig. 3C). However, both *Fis1* and *Mfn1* were not effectively upregulated in KO^IEC^ mice during DSS-induced colitis (Fig. 3C). Another key gene involved in fusion, *Mfn2*, was markedly decreased in KO^IEC^ mice after DSS treatment, whereas no obvious change occurred in WT mice (Fig. 3C). There were no significant differences in the expression of other mitochondrial dynamics-related genes (*Opa1* and *Dnm1l*), or *Pink1*, which is involved in mitophagy, between the four groups (Fig. 3C). In addition, a proteomics study was performed using the colon tissues of KO^IEC^ and WT mice after DSS treatment, and results of LC-MS/MS quantitative analysis showed significant differences in the protein expressions of *Mfn1*, *Fis1*, and *Dnm1l* between KO^IEC^ and WT group (Fig. S5A, B), indicating unbalanced mitochondrial dynamics after CKMT1 deletion. Of note, a dramatical increase of Fis1 protein in KO^IEC^ mice was observed both in proteomics and Western blotting (Fig. S5C), which was inconsistent with qPCR results. No significant difference regarding PGC-1α level, an indicator of mitochondrial biogenesis, was found between KO^IEC^ and WT group (Fig. S5D).

We also assessed the activity of the mitochondrial antioxidant system via qPCR. The colonic expressions of antioxidant genes, including *Prdx5*, *Gpx1*, and *Txnrd2*, were markedly upregulated after DSS exposure, indicating excessive oxidative stress during intestinal inflammation; however, no significant differences were found between the WT and KO^IEC^ groups (Fig. 3D). Moreover, although the expression of *Prdx3*, *Sod2*, and *Gpx4* did not significantly increased after DSS treatment in WT mice, we noted a marked reduction for *Prdx3* in KO^IEC^ mice (Fig. 3D), demonstrating a defect in antioxidant function. Western blotting also showed that most of the antioxidant proteins were increased upon DSS exposure in the colon of mice, but the expression of Prdx3 in KO^IEC^ mice failed to upregulated effectively during inflammation (Fig. 3E). Consistently, ROS production was obviously elevated in the colon tissue of KO^IEC^ mice compared to that in WT mice during DSS-induced colitis (Figs. 3F and S5E).

To further investigate the effect of CKMT1 on mitochondrial function, we measured the mitochondrial membrane potential (MMP) of the NCM460 cells with CKMT1 overexpression (CK^OE^) or control (NC) (Fig. S5F, G), using JC-1 probe. After 24 h TNF-α treatment, the MMP of CK^OE^ cells was apparently higher than that of NC cells (Fig. S5G, H). Mitochondrial morphology revealed that NC cells tended to be more fragmented under stress, while CKMT1 overexpression significantly protected the typical elongated mitochondrial morphology (Fig. 3G).

The above results suggest that colonic mitochondrial dysfunction occurs in colitis or inflammatory model after CKMT1 deletion.

### CKMT1 expression protects IECs from apoptosis

Mitochondrial dysfunction, especially oxidative stress, plays a major role in apoptosis [21]. Therefore, we asked whether apoptosis increased in KO^IEC^ mice, potentially accounting for the aggravated inflammatory phenotype, since IEC apoptosis is well recognized as a driver of intestinal inflammation [22]. As expected, TUNEL staining revealed increased colonic apoptosis in KO^IEC^ mice (Figs. 3H and S5I). Similarly, Western blotting revealed that during DSS-induced colitis, the protein expression of cleaved-caspase 3 was significantly elevated in the colon tissue of KO^IEC^ mice compared to that in WT mice (Fig. S6A).

**Fig. 4:**
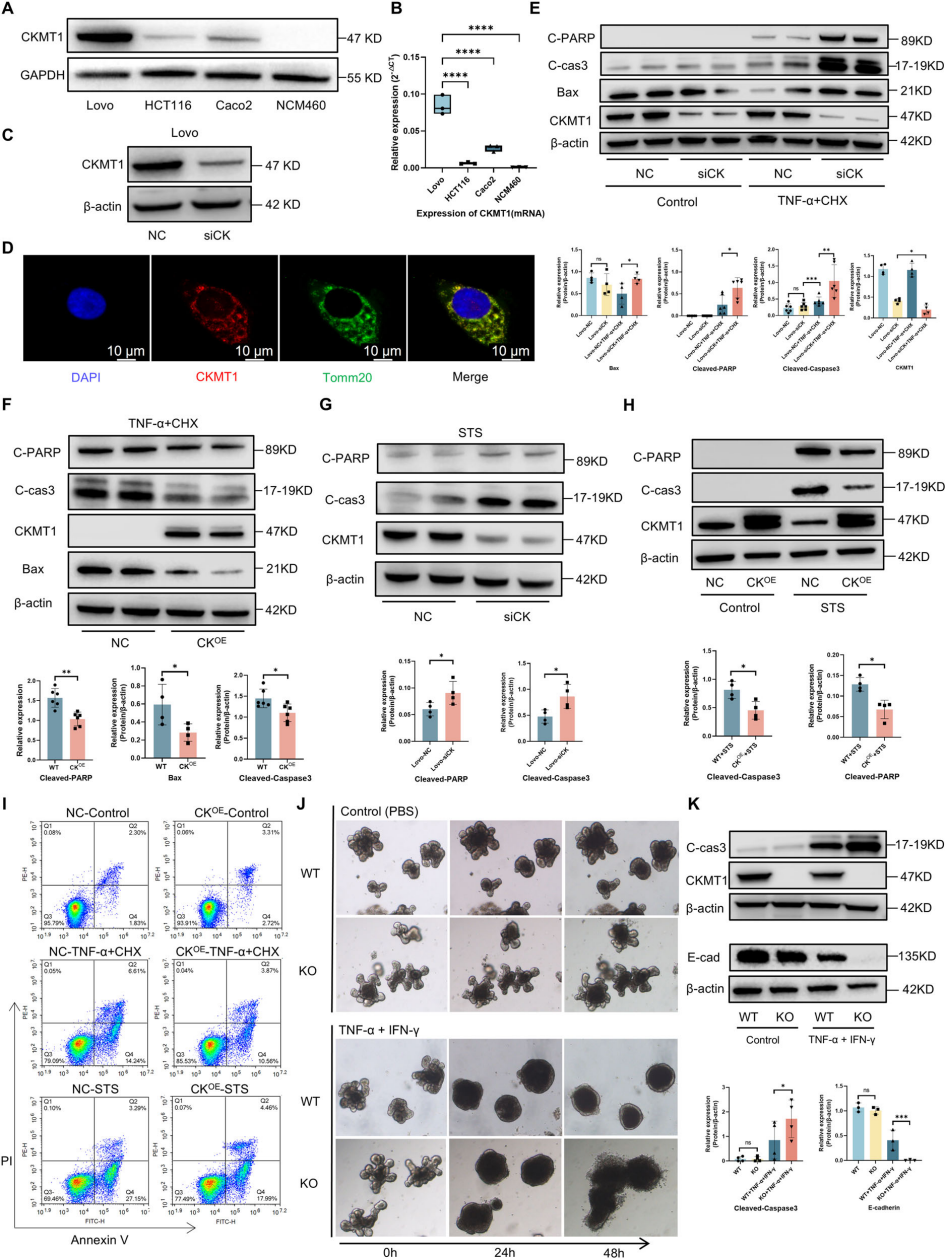
CKMT1 regulated both intrinsic and extrinsic apoptotic pathways in IECs in vitro. **A** The protein and **B** transcriptional expression of CKMT1 in Lovo, HCT116, Caco2, and NCM460 (n = 3). **C** The CKMT1 knockdown in Lovo were verified via Western blotting. **D** Confocal microscopy showing the co-localization of CKMT1 and Tomm20 (a marker of mitochondria) protein in IECs. After stimulated with TNF-α (50 ng/ml) and CHX (20 μg/mL) for 4 h, expressions of apoptosis-related proteins were assessed via Western blotting, **E** in Lovo cells with CKMT1 knockdown (siCK) or control (NC), and **F** NCM460 cells (n = 4–6). After stimulated with STS (2 μM) for 4 h, apoptosis-related proteins expressions were assessed via western blotting in **G** Lovo cells, or **H** NCM460 cells (n = 4). **I** Apoptosis levels of NCM460 cells after apoptotic stimulation were assessed with Annexin V-FITC/propidium iodide (PI) staining by flow cytometry (n = 3). **J** Representative images showing drastic change in morphology of intestinal organoid from KO^IEC^ or WT mice, after TNF-α (50 ng/ml) and IFN-γ (50 ng/ml) treatment. **K** Western blotting showing the expressions of cleaved caspase 3 (n = 4) and E-cadherin (n = 4) in organoids (harvested at 48 h after treatment). *P < 0.05; **P < 0.01; ***P < 0.001.

To exclude potential interference from other cell types, we examined the effect of CKMT1 on IEC apoptosis using IEC lines. Firstly, we generated CKMT1 stable knockdown cells (abbreviated as siCK) using Lovo cells, which demonstrate relatively high CKMT1 expression among several common IEC lines (Fig. 4A–C). Mitochondrial localization of CKMT1 protein was also confirmed (Fig. 4D). Subsequently, we tested the sensitivity of the cells to apoptosis. TNF-α, the critical proinflammatory mediator of IBD, combined with cycloheximide (CHX), was used to induce apoptosis [15, 23]. Immunoblot analysis revealed a significant increase in the expression of apoptosis-related markers, including cleaved poly (ADP-ribose) polymerase (cleaved-PARP), cleaved-caspase 3, and Bax in the siCK group after an apoptotic stimulus (Fig. 4E). To further confirm the above result and rule out the possible effect of tumor properties of Lovo cells on apoptosis, we validated above results via overexpressing CKMT1 in NCM460 cells, a normal colon epithelial cell line (Fig. S5F). As expected, compared with the NC group, the overexpressing group exhibited markedly reduced TNF-α-induced apoptosis (Fig. 4F). Given that TNF-α commonly activates the extrinsic apoptotic pathway, we investigated whether intrinsic apoptotic pathway was involved in CKMT1-mediated apoptosis-resistance. Thus, we treated siCK or CK^OE^ cells with staurosporine (STS) to induce intrinsic apoptosis and found that CKMT1 also played a protective role in intrinsic pathway-induced apoptosis (Figs. 4G, H). Apoptosis levels measured by flow cytometry further confirmed the above results (Fig. 4I). Moreover, we observed that intestinal organoids derived from KO^IEC^ mice disintegrated faster than WT group upon pro-inflammatory factors treatment (Fig. 4J), and immunoblot analysis also revealed increased apoptosis, as well as compromised barrier function (E-cadherin) in KO^IEC^ organoids (Fig. 4K).

Together, these findings indicate that CKMT1 expression limits the activation of both intrinsic and extrinsic apoptotic pathways in IECs.

### Loss of CKMT1 increases mitochondrial ROS via RET

**Fig. 5:**
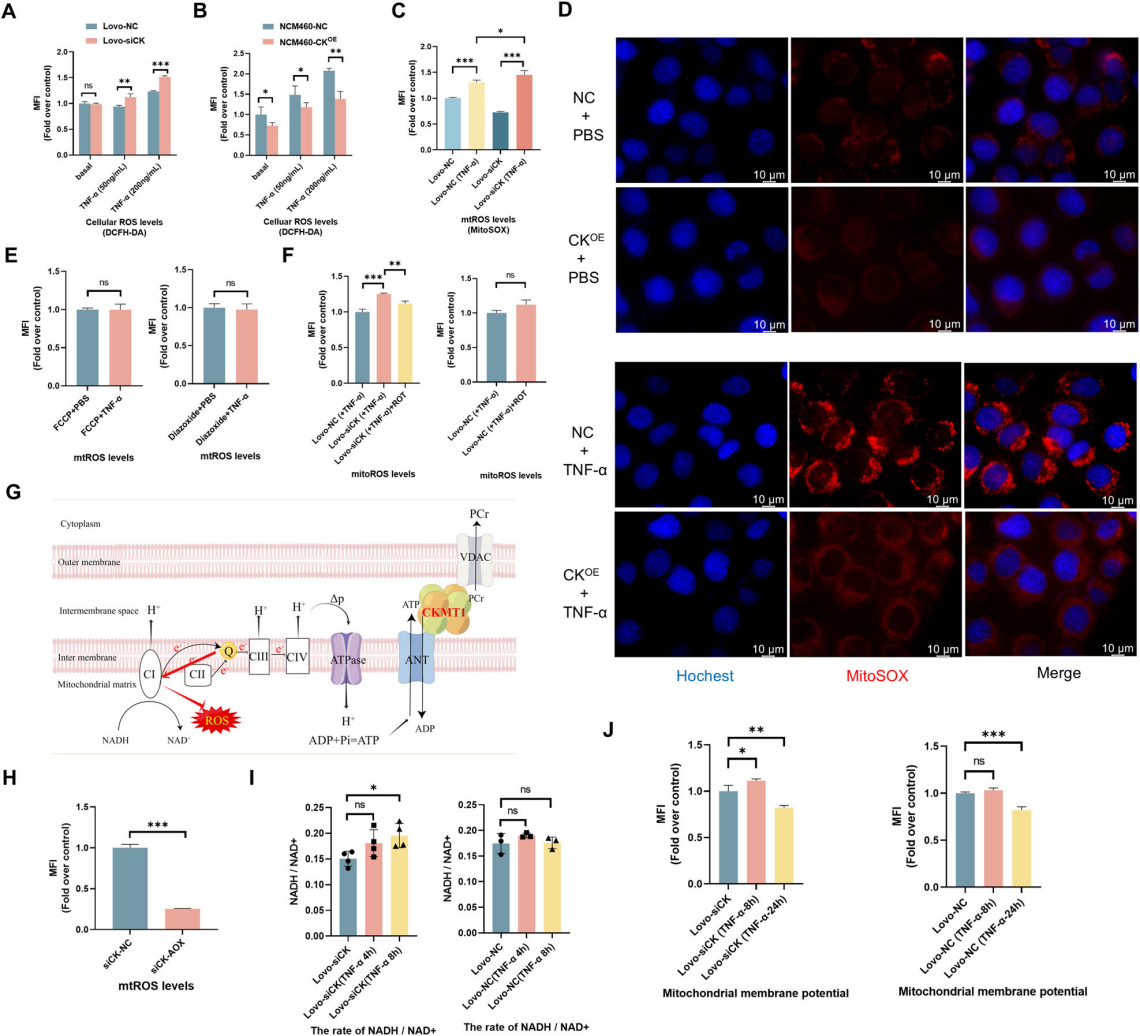
CKMT1 deficiency increased mitochondrial ROS via RET. After treated with TNF-α for 24 h (50 ng/ml), DCFH-DA staining showing cellular ROS levels in (**A**) Lovo cell, or (**B**) NCM460 cells, via flow cytometry. After treated with TNF-α for 24 h, MitoSOX staining of mitochondrial ROS (mtROS) was assessed via **C** flow cytometry in Lovo cells, or **D** via fluorescence microscopy in NCM460 cells. **E** The effects of FCCP (100 nM) or diazoxide (200 nM) pretreatment on TNF-α-induced mtROS in siCK Lovo cells were assessed via flow cytometry. **F** The mtROS levels were measured by flow cytometry in Lovo cells pretreated with rotenone (10 nM). **G** Illustration of electron transport chain-derived ROS production at complex I via RET. **H** AOX expression markedly reduced the TNF-α-induced mtROS levels in siCK cell. **I** NADH/NAD^+^ ratio was measured in cell lysis via microplate reader (n = 3 or 4), and **J** MMP (stained with TMRE) was measured via flow cytometry. All flow cytometry experiments were repeated three times. *P < 0.05; **P < 0.01; ***P < 0.001.

Mitochondria play a central role in the intrinsic apoptotic pathway (also known as the mitochondrial pathway) [24]. Interestingly, the extrinsic apoptotic pathway could also converge at mitochondria in the case of BID activation [25, 26]. Thus, we focused our subsequent studies on mitochondria to explain how CKMT1 regulates the apoptotic process. As demonstrated in the previous section, ROS, the potent inducer of apoptosis mainly produced in mitochondria [27], were excessively generated after CKMT1 deletion in colitis (Fig. 3F). Therefore, we assessed cellular ROS levels with DCFH-DA in an in vitro inflammation model induced by TNF-α at different concentrations and found that ROS were noticeably increased in Lovo cells with CKMT1 knockdown (Fig. 5A). Moreover, compared with the NC group, the CKMT1-overexpressing group exhibited significantly reduced TNF-α-induced ROS generation (Fig. 5B). As DCFH-DA indicates total cellular ROS, we then used a mitochondrion-targeted ROS probe (MitoSOX) to detect mitochondrial ROS (mtROS), and obtained similar results by flow cytometry (Fig. 5C and S6B) and fluorescence microscopy (Figs. 5D and S6C).

Although the electron transport chain (ETC) is the major source of mtROS, other mitochondrial enzymes, such as NOX4, p66Shc, and MAO-A/B, also contribute to mtROS generation [28]. We further confirmed that the increase in TNF-α-induced mtROS in IECs with CKMT1 knockdown was derived from the ETC, because the effect could be eliminated by two ETC inhibitors, FCCP or diazoxide (Fig. 5E). ETC derived mtROS are mainly generated at complex I when the leaked electron is transferred to O_2_ [29]. Forward electron transfer (FET) and reverse electron transport (RET) are two distinct mechanisms involved in the above process [29]. To determine the specific mechanism, we blocked the I_Q_ site of complex I with rotenone, which reduces mtROS from RET but not FER [29, 30]. Flow cytometry analysis revealed that rotenone pretreatment significantly reduced TNF-α-induced mtROS in cells with CKMT1 knockdown, but raised mtROS in NC cells, although the difference did not reach significance (*P* = 0.054) (Fig. 5F). Interestingly, as a kinase capable of catalyzing mitochondrial ATP generated by oxidative phosphorylation, CKMT1 is obviously functionally coupled with the ETC (Fig. 5G). Thus, the increase in TNF-α-induced mtROS in siCK cells likely originated from the ETC via the RET mechanism.

ROS generation via RET is driven by a highly reduced coenzyme Q (CoQ) pool and a high proton motive force (Δp) [31]. Alternative oxidase (AOX) can transfer electrons from CoQH_2_ to O_2_ (skipping complexes III and IV), thus preventing the overreduction of the CoQ pool and subsequent generation of mtROS via RET [32]. We expressed Ciona intestinalis AOX in siCK Lovo cells, and found that AOX expression obviously decreased TNF-α-induced mtROS compared to that in NC cells (i.e., siCK cells) (Fig. 5H). The NADH/NAD^+^ ratio is another marker of RET because a high Δp can reduce NAD^+^ to NADH. We detected an increased NADH/NAD^+^ ratio in siCK cells at an early time point after TNF-α treatment (Fig. 5I). Besides, ROS production via RET strongly depends on high MMP, which, together with the pH gradient constitute Δp [33]. Consistently, at 8 h after TNF-α treatment, we observed a significant increase of the MMP in siCK cells, but not in NC cells (Fig. 5J). These results further supported the conclusion that CKMT1-mediated mtROS originates from RET.

Collectively, our data suggested that loss of CKMT1 in IECs promotes TNF-α-induced mtROS generation via the RET pathway.

### ROS-induced mitochondrial permeability transition pore (mPTP) opening contributes to CKMT1-mediated apoptosis

**Fig. 6:**
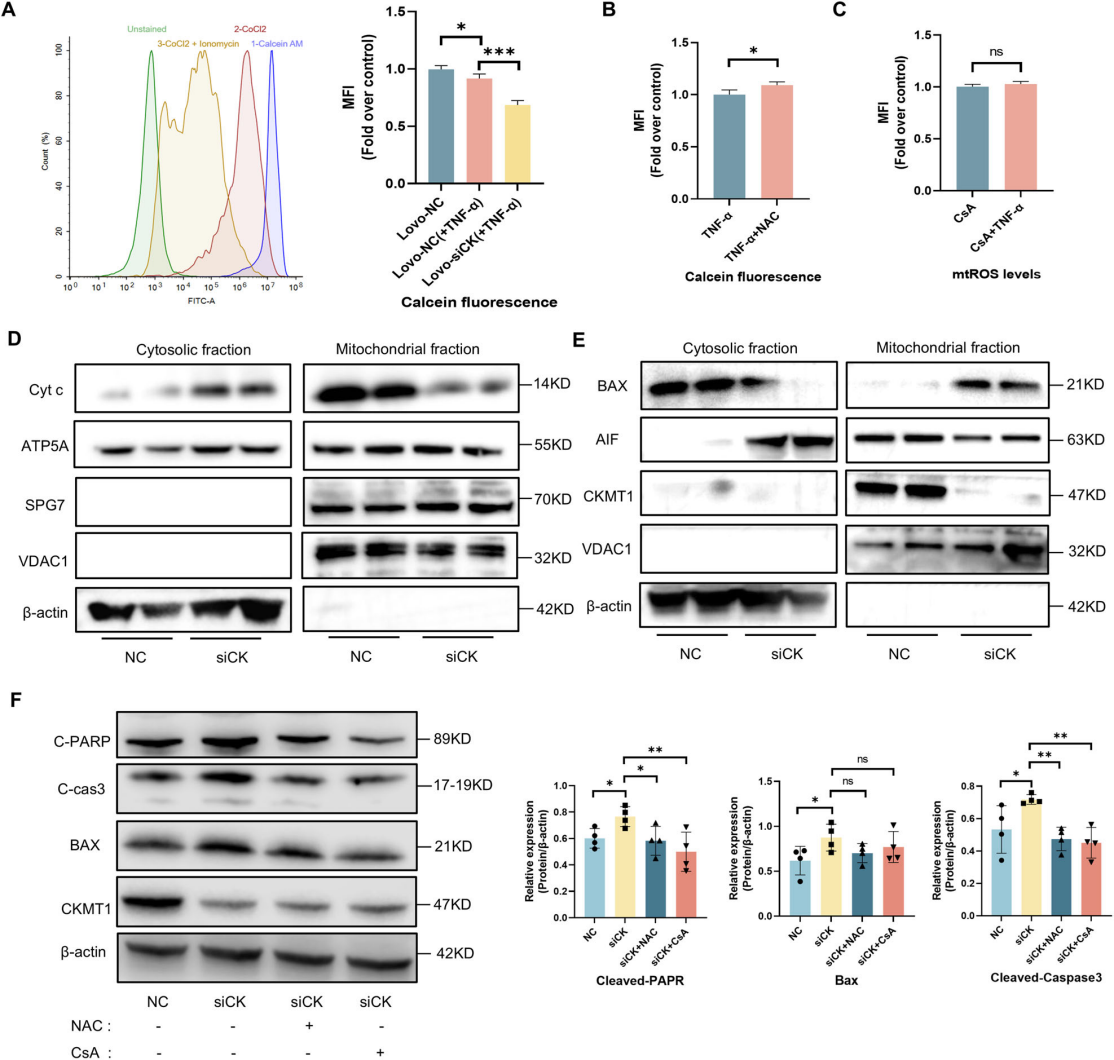
ROS and mPTP were involved in CKMT1-mediated IECs apoptosis. **A** Flow cytometry analysis of the opening of mPTP (Calcein fluorescence) in Lovo cells. **B** Flow cytometry analysis of the effect of NAC pretreatment (5 mM for 3 h) on the opening of mPTP induced by TNF-α in siCK cells. **C** Flow cytometry analysis of the effect of CsA pretreatment (1 μM for 3 h) on TNF-α-induced mtROS generation in siCK cells. **D** Assessment of cytochrome c, ATP5A, and SPG7 in cytosolic or mitochondrial fraction of siCK cells after apoptosis induction via Western blotting. **E** Assessment of AIF and BAX expressions after apoptosis induction via Western blotting. **F** Assessment of the levels of apoptosis-related proteins in siCK cells upon NAC and CsA pretreatment via western blotting (n = 4). All flow cytometry experiments were repeated three times. *P < 0.05; **P < 0.01; ***P < 0.001.

ROS are the most frequently mentioned inducers of the mPTP, and opening of the mPTP may cause a dramatic fall of MMP [34]. It explained the apparent decrease of MMP in IECs at 24 h after TNF-α treatment (Fig. 5J), which is usually considered an early hallmark event of apoptosis. We then measured the mPTP in IECs with Calcein-CoCl_2_ technique via flow cytometry (Fig. 6A). TNF-α triggered more extensive opening of the mPTP in the siCK group than in the NC group (Figs. 6A and S6D), whereas scavenging ROS with N-acetylcysteine (NAC) markedly reduced mPTP opening (Fig. 6B). The opening of mPTP opening has been reported to promote further generation of ROS [35], thus likely establishing a vicious cycle in IECs with CKMT1 deficiency during inflammation. Indeed, TNF-α failed to increase mtROS levels in siCK cells in the presence of Cyclosporin A (CsA), an inhibitor of mPTP (Fig. 6C). These data demonstrated a close interrelationship between RET-ROS and the mPTP.

Excess opening of the mPTP causes swelling of the mitochondrial matrix and mitochondrial outer membrane permeabilization (MOMP), resulting in the release of cytochrome c from the mitochondria to the cytoplasm, which ultimately stimulates caspase activation and apoptosis [36]. To further verify our hypothesis, mitochondria were isolated for cytochrome c analysis. As expected, significant increased cytochrome c in the cytoplasmic fraction was observed after exposure to the apoptotic stimulus (TNF-α + CHX) in the siCK group compared to that in the NC group (Fig. 6D). A similar trend was also observed for another proapoptotic protein, apoptosis-inducing factor (AIF) (Fig. 6E). Conversely, we found that cytosolic Bax was recruited to the mitochondria (Fig. 6E), which is crucial for MOMP and apoptosis. In addition, two newly identified mPTP components [34], SPG7 and ATP5A, were evaluated, but no obvious change was detected (Fig. 6D). Despite this, the above data still implied that the mPTP is likely crucial in CKMT1-mediated apoptosis.

At last, we pretreated cells with NAC or CsA before inducing apoptosis. Western blotting revealed significantly lower levels of cleaved PARP and cleaved caspase 3 in the siCK cells pretreated with NAC or CsA than those in the siCK cells (Fig. 6F). A similar trend was observed for Bax expression; however, it did not reach significance in the multiple comparison post hoc analysis (Fig. 6F). NAC or CsA pretreatment also delayed the rapid structural disintegration of intestinal organoids from KO^IEC^ mice. (Fig. S6E). These data demonstrated that scavenging ROS and inhibiting mPTP opening can rescue the apoptotic phenotype of IECs caused by decreased CKMT1 expression.

Thus, we concluded that RET-ROS and ROS-induced mPTP opening are responsible, at least in part, for IEC apoptosis in CKMT1-deficient cells.

## Discussion

IECs apoptosis and oxidative stress have long been recognized as important causes and features of IBD [22, 37]. Nowadays, numerous studies have increasingly confirmed that mitochondrial dysfunction is an important cause of IBD [5]. These features were confirmed in our study as well (Fig. S4). Meanwhile, IBD, especially UC, has also been considered an energy deficient disease of the intestinal tract [38, 39]. In a recent large UC transcriptomic cohort, all 13 genes from mitochondrial genomes were significantly downregulated in patients with active UC. Notably, these genes encode crucial mitochondrial proteins that act as electron transport chain components (including Complexes I, III, IV, and V), playing a central role in energy production during oxidative phosphorylation [40]. However, despite their key roles in energy metabolism, CKs in intestinal inflammation have received insufficient attention. Colgan et al [13]. previously explored the function of cytosolic CKs in colitis and revealed that HIF-regulated CK controls IECs homeostasis and the mucosal barrier. This group recently reported that mice with CKs (brain and mitochondrial type) global deletion exhibited increased susceptibility to colitis due to the loss of IFN-γ production [41]. However, to date, no in-depth exploration of the role of mitochondrial CK in IBD has been conducted. In our study, we demonstrated that CKMT1 protein level in inflammatory colon tissue was markedly reduced. By establishing intestinal epithelial-specific CKMT1 knockout mice and an IBD mouse model, we comprehensively examined the colitis phenotype and mitochondria-associated phenotypes, such as mitochondrial homeostasis, oxidative stress, and apoptosis. Our findings revealed a key role for CKMT1 in maintaining intestinal homeostasis and mitochondrial function.

To explain how CKMT1 influences intestinal inflammation, we focused on apoptosis, a major consequence of mitochondrial dysregulation and an important contributor to inflammation. Our in vitro experiment demonstrated that CKMT1 is crucial for controlling IEC apoptosis by limiting the activation of both intrinsic and extrinsic apoptotic pathways. Next, we further explored the underlying mechanism involved. Previous evidence has suggested that mitochondrial CK expression could reduce mtROS in the heart, liver, or brain [17, 42]. Consistently, we confirmed that CKMT1 also regulated mtROS in IECs during colitis. Mechanistically, we demonstrated that the RET pathway is involved in CKMT1-mediated mtROS production and the RET-ROS contributes to mPTP opening and subsequent apoptosis in IECs. Although RET was considered an in vitro artifact in the past and has never been reported in IBD, its pathological role in other diseases, such as cardiac ischemia-reperfusion injury [43] and tuberculosis [44], has recently been well established. CKMT1 located in the mitochondrial intermembrane space (IMS) and is spatially close to the adenine translocator [45], which is responsible for transporting ATP from the mitochondrial matrix to the IMS. This special location doubtlessly facilitates the function of the PCr/CK system. Thus, in theory, CKMT1 located downstream of the ETC both positionally and functionally (Fig. 5G). In the present study, we speculated that loss of the CKMT1 protein (or function) may slow ATP generation from ATP-synthase via a certain feedback mechanism (e.g., accumulated local ATP in the IMS), and subsequently drive RET through upstream overproduced Δp. However, further investigation is needed to clarify the molecular mechanism underlying CKMT1-mediated RET-ROS generation and to validate the RET mechanism in vivo.

Notably, our findings may signify novel therapeutic directions for colitis treatment. For example, approaches that target mitochondrial function, such as mitochondrial dynamics [46] or biogenesis [47], may have therapeutic potential in IBD. Treatment of chow supplemented with Cr (substrate of CKs) markedly alleviated DSS-induced colitis in mice [13]. Furthermore, the efficacy of mtROS-targeted scavenger therapy (MitoQ) for adults or children with UC is being evaluated in two ongoing clinical trials (ClinicalTrials.gov Identifier: NCT05539625, NCT04276740).

In summary, CKMT1 is important for maintaining mitochondrial homeostasis and regulating mtROS generation in IECs. Loss of the CKMT1 protein may contribute to colitis pathogenesis via RET-ROS/mPTP pathway-mediated apoptosis. Our data provide a new perspective on the role of CKMT1 and RET-ROS in colitis.

## Materials and methods

### Human specimens

UC patients and healthy volunteers were recruited from the Gastroenterology Department of Shanghai Changhai Hospital. Colon tissues were obtained by endoscopic biopsy. Ethical approval was obtained from the Institutional Review Board and Ethics Committee of Shanghai Changhai Hospital (No. CHEC2021-029). All individuals signed informed consents documentation and the study was conducted in accordance with the principles of the Helsinki Declaration.

### Mice

The generation of intestinal epithelial-specific *Ckmt1* conditional knockout (*Ckmt1*^flox/flox, Vil-Cre^) mice (C57BL/6J) was assisted by Cyagen Biosciences (Suzhou, China) through CRISPR/Cas-mediated genome engineering. All mice were housed at the Animal Research Center of Changhai Hospital under specific pathogen-free conditions. Cre-negative *Ckmt1*^flox/flox^ littermates were used as controls. Sex-matched and age-matched (aged 8–16 weeks) mice were used for each experiment. All animal experiments were reviewed and approved by the Committee on Ethics of Medicine of Navy Medical University, Shanghai, China.

### Experimental colitis model

Wild-type mice were administered 3% (w/v) DSS (MP Biomedicals) in drinking water for 7 days to induce acute colitis. *Ckmt1*^flox/flox, Vil-Cre^ mice and their littermate controls were fed 2% (w/v) DSS for 8 or 9 days. Weight loss, stool consistency, and occult or gross blood presence were recorded daily to calculate the disease activity index (DAI) [48]. Mice were sacrificed by cervical dislocation, and the colon was removed and flushed with cold PBS. Unless indicated otherwise, distal colonic tissues were collected for further experiments, such as Western blotting and tissue sections.

### Cell and organoid culture

Cells (NCM460, Lovo, Caco2, and HCT116) and organoids were cultured in a cell culture incubator under standard culture conditions (37°C, 5% CO_2_). Lovo cells were maintained in Ham’s F-12K medium (Pricella, China), and the other cell lines were maintained in Dulbecco’s Modified Eagle Medium (DMEM; Gibco, USA), both of which were supplemented with 10% fetal bovine serum (FBS; Gibco, USA) and 1× penicillin/streptomycin. Organoids from the mouse small intestine were isolated and cultured as described previously [49]. Cells were infected with lentivirus containing shRNA (CKMT1A) or overexpression (CKMT1A or Alternative oxidase) constructs generated by OBiO Technology (Shanghai, China) to produce knockdown or overexpression stable cell lines; antibiotic resistance screening was used for selection. Further details are presented in the Supplementary Methods.

### Western blotting

Cell or tissue proteins were extracted with RIPA buffer (Beyotime, China) supplemented with protease inhibitors (Epizyme, China). Total proteins were separated on 4–20% SDS-polyacrylamide gels (GenScript, China) and transferred to PVDF membrane (Millipore, USA). After blocking with 5% nonfat milk in Tris-buffered saline with Tween (TBST), the membrane was incubated overnight with the primary antibody at 4 °C on a shaker. Subsequently, the membrane was washed with TBST and incubated with horseradish peroxidase-labeled secondary antibody for 1 h at room temperature. After the final washing step, the membrane was visualized with an enhanced chemiluminescence detection kit (Epizyme, China) in an Amersham 600 imager (GE Healthcare, USA) or Odyssey scanner (LI-COR, USA).

### Quantitative real-time PCR (qPCR)

Total RNA was extracted from colon tissue or cells with a Total RNA Isolation Kit (Vazyme, China) and immediately reverse transcribed into cDNA with PrimeScript RT Master Mix (Takara, Japan). qPCR was conducted on a LightCycler 480 II (Roche, Switzerland) instrument using a TB Green Premix Ex Taq™ II kit (Takara, Japan) according to the manufacturer’s instructions. In DSS-induced colitis of mice, TATA-box-binding protein (*Tbp*) was used as an internal reference gene for the normalization of colonic target genes [50]. GAPDH was selected as the internal reference gene in other contexts.

### Detection of ROS

For total cellular ROS detection, approximately 2 × 10^6^ cells were harvested and incubated (20 min, 37 °C, light avoidance) with 2,7-dichlorofluorescein diacetate (DCFH-DA, Beyotime) at working concentrations (1:1000 dilution). For mitochondrial ROS detection, cells were harvested and incubated (30 min, 37 °C, light avoidance) with 500 nM MitoSOX™ Red (Invitrogen, USA). Cells were gently washed 3 times with prewarmed HBSS (with calcium and magnesium), and ROS levels were determined immediately via NovoCyte flow cytometry (Agilent, USA). For in situ mitochondrial ROS detection, cells, cultured in confocal dishes, were incubated with MitoSOX™ Red (500 nM, diluted in HBSS with calcium and magnesium) for 30 min and then Hoechst 33342 (MedChemExpress, USA) for 10 min (both at 37 °C, light avoidance). The cells were gently washed 3 times with prewarmed HBSS and immediately analyzed via fluorescence microscopy (Leica, Germany). For ROS detection in colon tissue, freshly frozen tissue sections were subjected to dihydroethidium (DHE) (Beyotime, China) staining. Briefly, colon sections were incubated with 5 μM DHE at 37 °C for 30 min (protected from light). After nuclear staining with DAPI for 10 min at room temperature, the sections were washed 3 times with PBS (PH 7.4) on a shaker. Finally, ROS levels were measured by fluorescence microscopy.

### Detection of mitochondrial membrane potential (MMP)

The MMP was measured with tetramethylrhodamine ethyl ester perchlorate (TMRE) (MedChemExpress, USA) according to the manufacturer’s instructions. Briefly, cells were harvested from 6-well plates and incubated (30 min, 37 °C, light avoidance) with 150 nM TMRE (diluted in 2 mL of HBSS containing calcium and magnesium). The cells were then gently washed three times with prewarmed HBSS, and the MMP was immediately analyzed via flow cytometry. Enhanced mitochondrial membrane potential assay kit with JC-1 (Beyotime, China) was also used to measure MMP. Briefly, cells were washed with prewarmed HBSS and incubated with JC-1 working solution (diluted in cell medium, 37 °C, light avoidance, 20 min). JC-1 dye buffer solution was then used to wash the cells. At last, the MMP was immediately analyzed via fluorescence microscopy.

### Statistics

Statistical analysis and data visualization were performed using SPSS (version 21.0) or GraphPad Prism (version 9.0). The results are shown as the mean ± SD. One-way ANOVA followed by the LSD test was used for multiple comparisons unless otherwise stated. Repeated measures ANOVA was used to compare changes in body weight loss across time among groups. Sample size calculation was not performed. Mice were allocated to experimental groups randomly. Investigator was not blinded to the group allocation. *P* < 0.05 was considered to indicate significance.

The enzyme-linked immunosorbent assay (ELISA), TUNEL staining, immunohistochemistry (IHC), IF, transmission electron microscopy, NADH/NAD^+^ quantification, detection of apoptosis (flow cytometry), mitochondrial permeability transition pore (mPTP) measurement, proteomics sequencing, Mitochondrial morphology imaging, and mitochondria isolation, protocol details are provided in the Supplemental Methods. The antibodies, qPCR primers, and other reagents used are listed in the Supplementary Tables.

## Supplementary information

supplementary materials

Original Data for Western Blotting

## Data Availability

The data are available from the corresponding author upon reasonable request and with the permission of the institution. The mass spectrometry proteomics data have been deposited to the ProteomeXchange Consortium via the PRIDE partner repository with the dataset identifier PXD057053.
