# Supplementary material for: CKMT1 deficiency contributes to mitochondrial dysfunction and promotes intestinal epithelial cell apoptosis via reverse electron transfer-derived ROS in colitis
Source: Cell Death Dis. 2025 Mar 15;16(1):177. doi: 10.1038/s41419-025-07504-4 (PMC11910573; doi:10.1038/s41419-025-07504-4)
Supplement: Supplementary file 1 — supplementary materials [file 41419_2025_7504_MOESM1_ESM.pdf]

Fig. S1

A

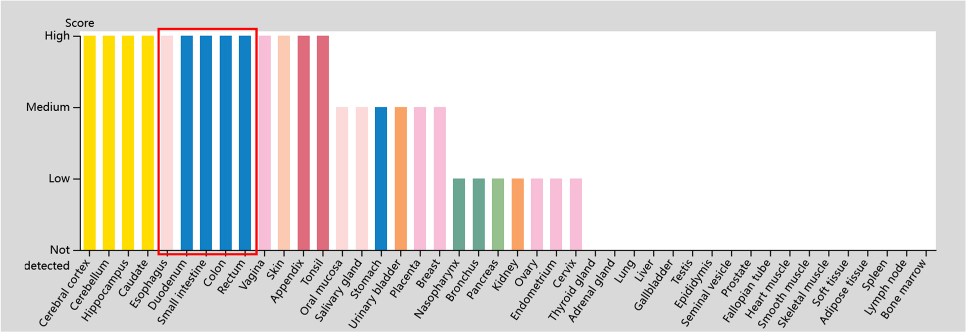

B

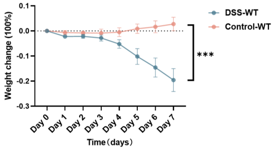

C

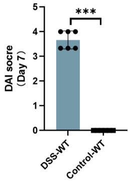

D

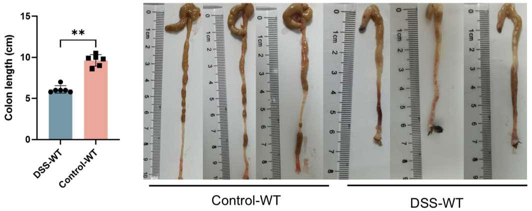

E

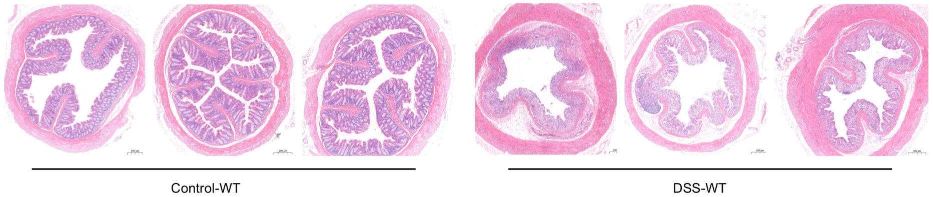

F

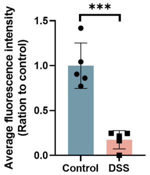

Fig. S2

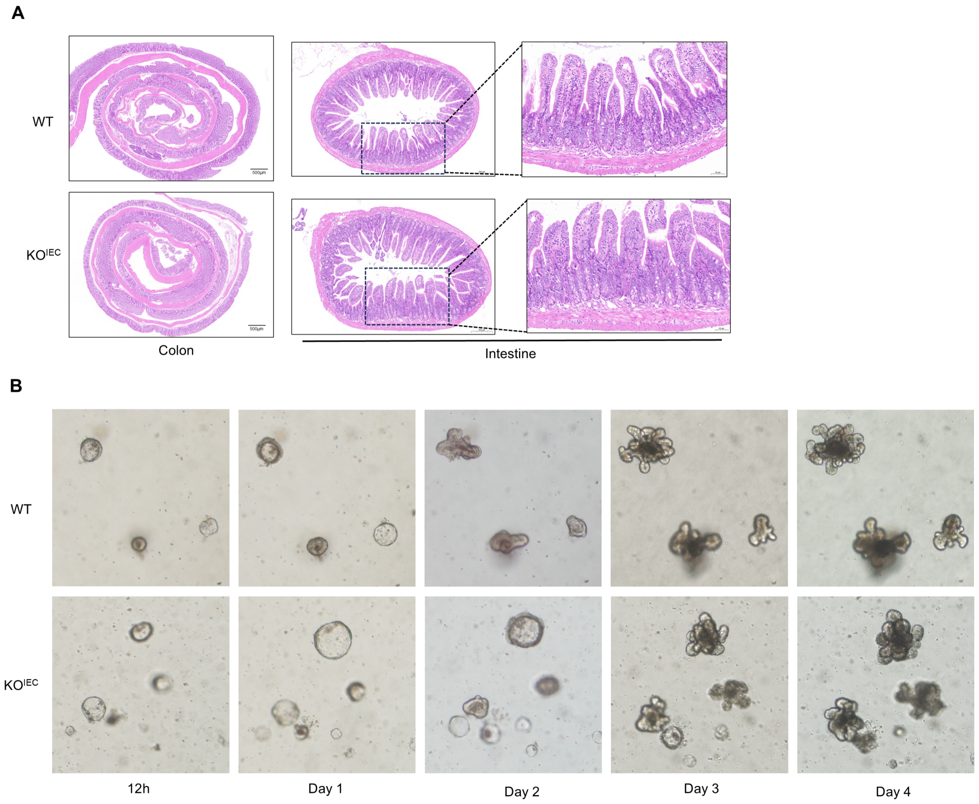

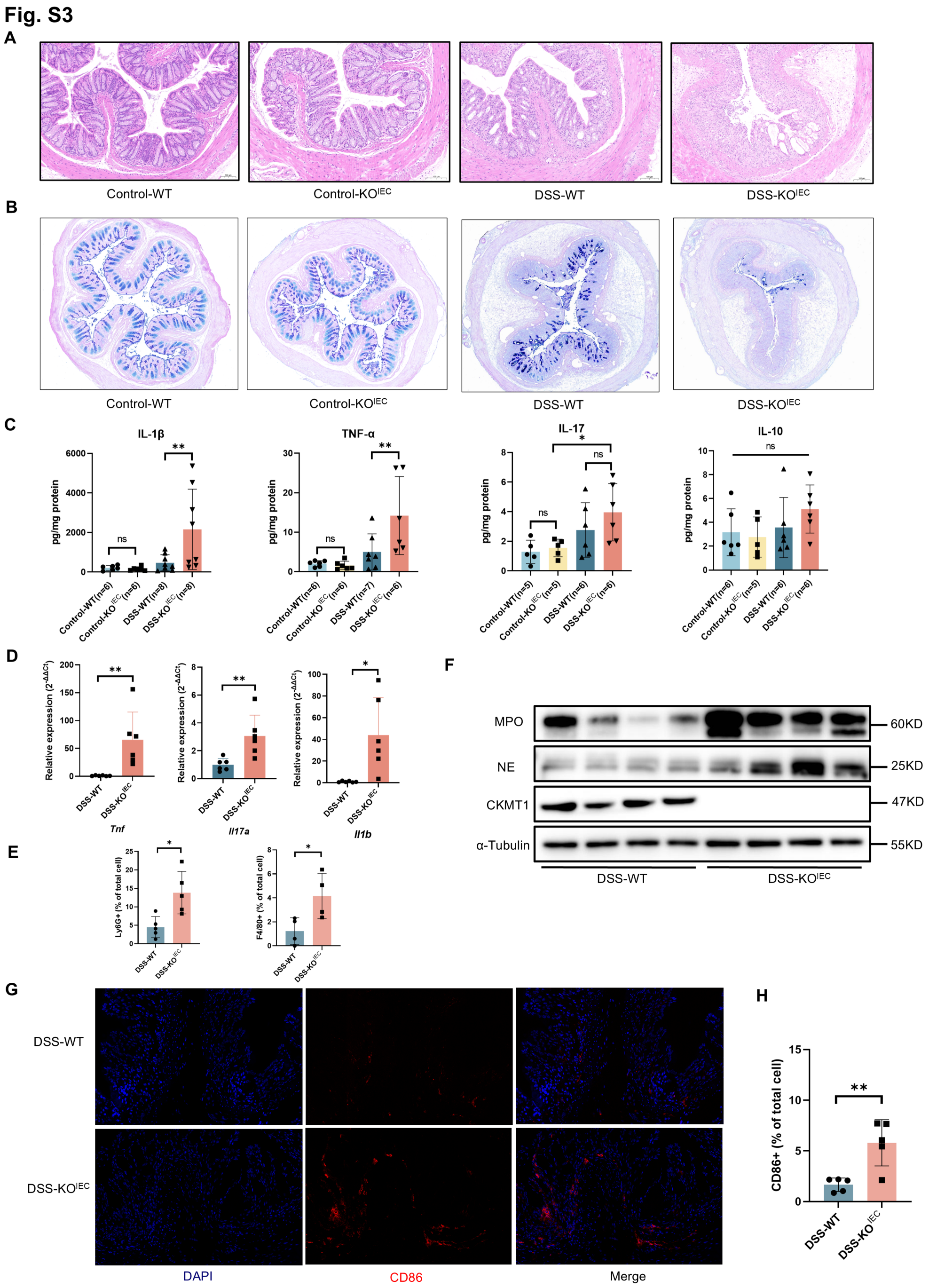
Fig. S3
A
Control-WT
Control-KO^IEC
DSS-WT
DSS-KO^IEC
B
Control-WT
Control-KO^IEC
DSS-WT
DSS-KO^IEC
C
IL-1β
TNF-α
IL-17
IL-10
pg/mg protein
ns
**
*
Control-WT(n=6)
Control-KO^IEC(n=6)
DSS-WT(n=8)
DSS-KO^IEC(n=8)
D
Relative expression (2^-ΔΔCt)
DSS-WT
DSS-KO^IEC
Tnf
Il17a
Il1b
E
Ly6G+ (% of total cell)
F4/80+ (% of total cell)
F
MPO
NE
CKMT1
α-Tubulin
60KD
25KD
47KD
55KD
DSS-WT
DSS-KO^IEC
G
DSS-WT
DSS-KO^IEC
DAPI
CD86
Merge
H
CD86+ (% of total cell)
**

Fig. S4

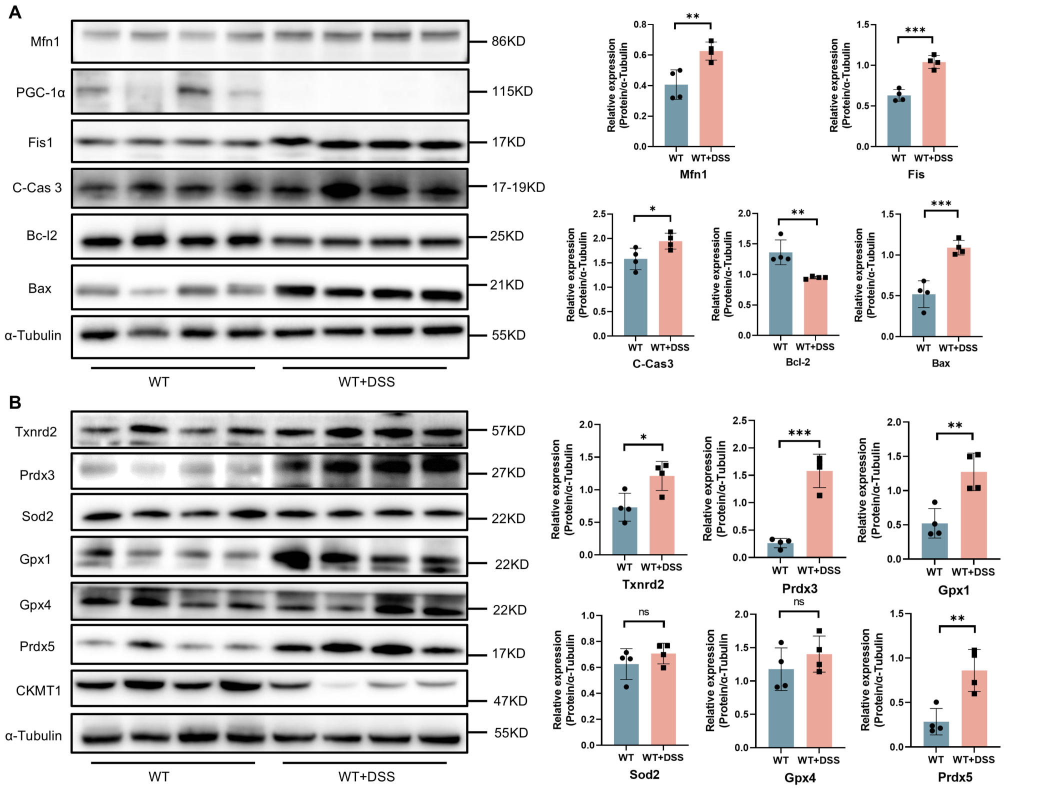
A
Mfn1
PGC-1α
Fis1
C-Cas 3
Bc-l2
Bax
α-Tubulin
86KD
115KD
17KD
17-19KD
25KD
21KD
55KD
WT
WT+DSS
Relative expression (Protein/α-Tubulin
Mfn1
Fis
C-Cas3
Bcl-2
Bax
B
Txnrd2
Prdx3
Sod2
Gpx1
Gpx4
Prdx5
CKMT1
57KD
27KD
22KD
47KD
Txnrd2
Prdx3
Gpx1
Sod2
Gpx4
Prdx5
ns

Fig. S5

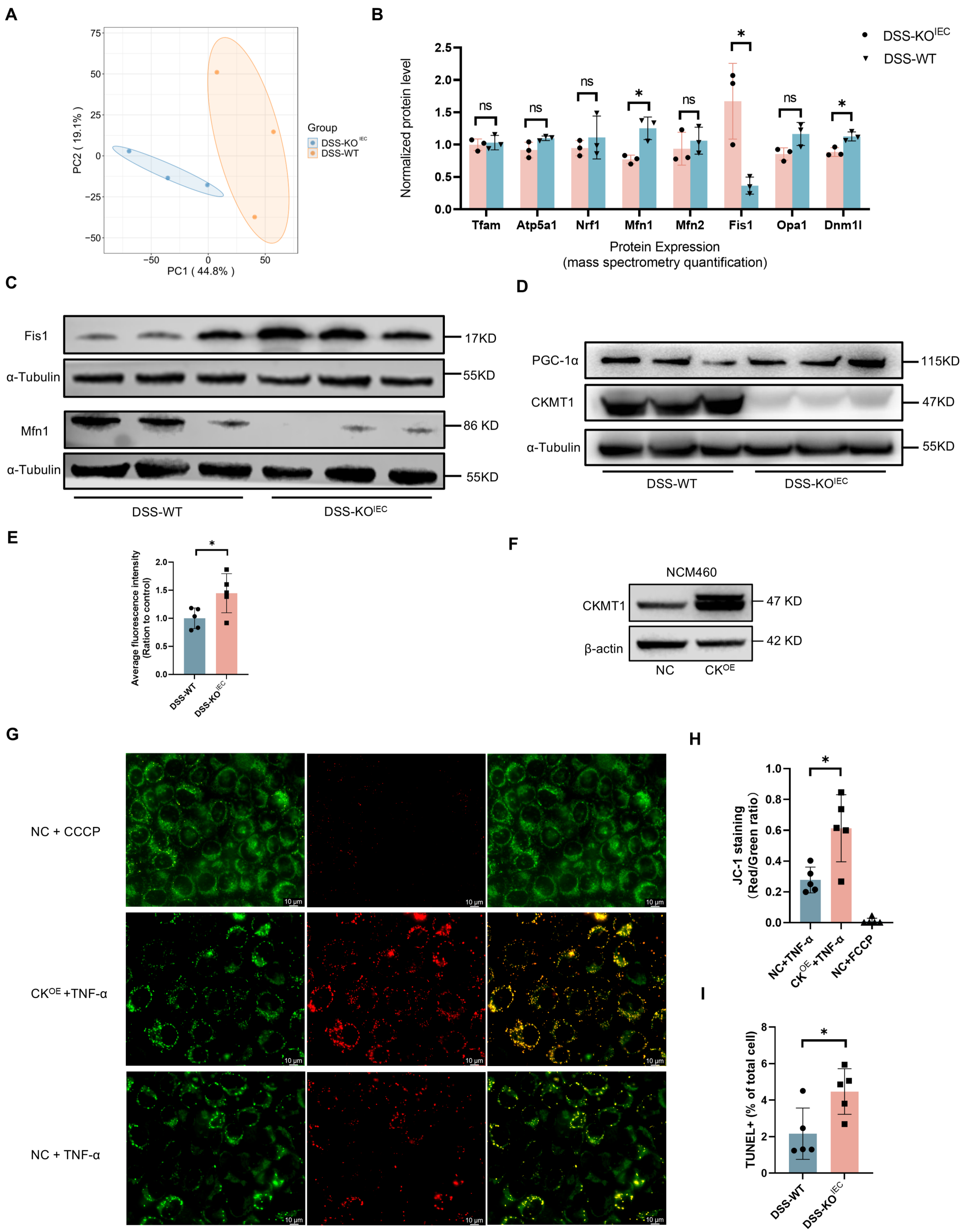

A
B
C
D
E
F
G
H
I
Group
DSS-KO IEC
DSS-WT
PC1 (44.8%)
PC2 (19.1%)
DSS-KO IEC
DSS-WT
Normalized protein level
Tfam
Atp5a1
Nrf1
Mfn1
Mfn2
Fis1
Opa1
Dnm1l
Protein Expression
(mass spectrometry quantification)
Fis1
α-Tubulin
Mfn1
α-Tubulin
17KD
55KD
86 KD
55KD
DSS-WT
DSS-KO IEC
PGC-1α
CKMT1
α-Tubulin
115KD
47KD
55KD
DSS-WT
DSS-KO IEC
Average fluorescence intensity (Ration to control)
DSS-WT
DSS-KO IEC
NCM460
CKMT1
β-actin
47 KD
42 KD
NC
CK OE
NC + CCCP
CK OE +TNF-α
NC + TNF-α
10 μm
JC-1 staining (Red/Green ratio)
NC+TNF-α
CK OE +TNF-α
NC+FCCP
TUNEL+ (% of total cell)
DSS-WT
DSS-KO IEC

Fig. S6

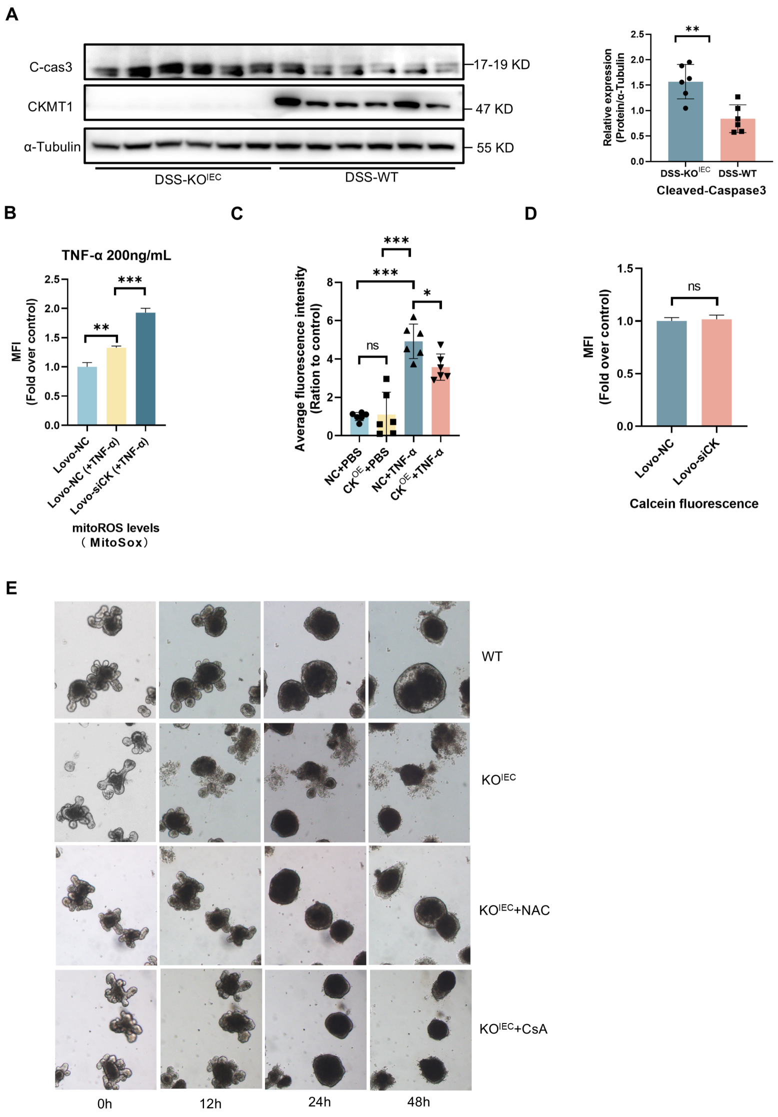
A
C-cas3
CKMT1
α-Tubulin
17-19 KD
47 KD
55 KD
DSS-KO^IEC
DSS-WT
Relative expression (Protein/α-Tubulin)
Cleaved-Caspase3
B
TNF-α 200ng/mL
MFI (Fold over control)
Lovo-NC
Lovo-NC (+TNF-α)
Lovo-siCK (+TNF-α)
mitoROS levels
（MitoSox）
C
Average fluorescence intensity (Ration to control)
NC+PBS
CK^OE+PBS
NC+TNF-α
CK^OE+TNF-α
ns
D
MFI (Fold over control)
Lovo-NC
Lovo-siCK
Calcein fluorescence
ns
E
WT
KO^IEC
KO^IEC+NAC
KO^IEC+CsA
0h
12h
24h
48h

Figure S1

(A) Protein expression (CKMT1A) overview among different human tissues from the Human Protein Atlas database (https://www.proteinatlas.org/). (B-E) Establishment of acute colitis model in wild-type mice by DSS. Weight curves (B), DAI scores at day 7 (C), colon length (D), representative colonic H&E staining (E) of mice treated with or without 3% DSS (n = 6). *, $P < 0.05$; **, $P < 0.01$; ***, $P < 0.001$. (F) Quantitative analysis of fluorescent intensity of CKMT1 in colon tissues (n = 3, 1–2 technical replicates pictures/mouse).

Figure S2

Epithelial CKMT1 depletion caused no obvious phenotype in mice. (A) H&E staining of colon and small intestine sections showing the intestine of CKMT1 KO^{IEC} mice (8 weeks old) developed normally without structural deficits. (B) Intestine organoids from KO^{IEC} mice developed normally (observed over 8 passages) (n = 3).

Figure S3

Epithelial CKMT1 deficiency aggravated DSS-induced colitis. (A-B) Representative images of (A) H&E staining, and (B) AB/PAS staining of colon tissues from KO^{IEC} and WT mice with or without DSS treatment. (C) ELISA assay (n = 5–8) for the protein levels IL-1β, TNF-α, IL-17, and IL-10, and (D) qPCR assessment (n = 6) for the mRNA levels of IL-1β, TNF-α, and IL-17 in colon tissues of mice. (E) Quantification of Ly6G+ or F4/80+ cells in colon tissues (n = 3, 1–2 technical replicates pictures/mouse). (F) Western blotting analysis for neutrophil elastase (NE) and myeloperoxidase (MPO) in colon tissues (n = 4). (G) Immunofluorescent staining for CD86 in the colon sections. (H) Quantification of CD86+ cells in colon tissues (n = 3, 1–2 technical replicates pictures/mouse) *, $P < 0.05$; **, $P < 0.01$; ***, $P < 0.001$.

Figure S4

Deterioration of mitochondrial dysfunction and IECs apoptosis during DSS-induced colitis. (A) Western blotting assessment for the protein expressions of mitochondrial function and apoptosis-related genes, and (B) mitochondrial specific ROS scavengers in colon tissues.

Figure S5

Epithelial CKMT1 deletion aggravated mitochondrial dysfunction. (A) Principal components analysis (PCA) on the proteomic data. (B) LC-MS/MS quantitative analysis (n = 3), and (C-D) Western blotting assessment for the levels of mitochondrial homeostasis-related proteins. (E) Quantitative analysis of ROS (DHE staining) fluorescent intensity in colon tissues (n = 3, 1–2 technical replicates pictures/mouse). (F) The CKMT1 overexpression in NCM460 were verified via Western blotting. (G) JC-1 staining (JC-1 monomers emit green fluorescence and JC-1 monomers emit green fluorescence) showing that the membrane mitochondrial potential (MMP) of NCM460 cells after 24 h TNF-α treatment (FCCP was used as the positive control). (H) Quantitative analysis of JC-1 fluorescent intensity (n = 3, with 1–2 random fields of view/picture). (I) Quantification of TUNEL+ cells in colon tissues (n = 3, 1–2 technical replicates pictures/mouse). *, $P < 0.05$; **, $P < 0.01$; ***, $P < 0.001$.

Figure S6

(A) Western blotting showing the expressions of cleaved caspase 3 in colon tissues from WT or KO^{IEC} mice after DSS administration (n = 6). (B) Flow cytometer showing increased mtROS levels in siCK cells (compared to that in NC cells) after a high concentration of TNF-α (200 ng/ml) treatment. (C) Quantitative analysis of mtROS fluorescent intensity of cells (n = 3, with 2 random fields of view/picture). (D) Knockdown of CKMT1 in Lovo cells did not induce mPTP opening under basal conditions (without TNF-α). (E) NAC or CsA pretreatment delayed the rapid structural disintegration of intestinal organoids from KO^{IEC} mice. All flow cytometry experiments were repeated three times. *, $P < 0.05$; **, $P < 0.01$; ***, $P < 0.001$.

Supplementary Methods

Cell culture

Cells were obtained from the National Collection of Authenticated Cell Cultures (Shanghai, China).

For CKMT1 knockdown, CKMT1A (NM_001015001.2) shRNA (shRNA1 target: 5′-ACCTGGCTGGACGTTACTATA-3′, shRNA2 target: 5′-AGGCGGTGTCTTTGAT-ATTTC-3′, shRNA3 target: 5′-CATCGATGGAGTAAACTATTT-3′, NC target: 5′-CCTAAGGTTAAGTCGCCCTCG-3′) were designed and cloned into the vectors (pSLenti-U6-shRNA-CMV-BSR-WPRE). Among them, lentivirus with shRNA1 was proved by qPCR to be more efficacious for CKMT1 knockdown in Lovo cells and thus used to generate stable cell lines.

For CKMT1 overexpression, full-length CKMT1A (NM_001015001.2) open reading frame was cloned into the vector (pLenti-CMV-MCS-HA-PGK-BSR-WPRE) using EcoRI and XbaI restriction enzyme sites (sequencing primers, Forward: 5′-CGCAAATGGGCGGTAGGCGTG-3′, Reverse: 5′-ggatgtggaatgtgtgcgaggc-3′), and then CKMT1A was overexpressed in NCM460 via lentivirus transfection.

For alternative oxidase (AOX) overexpression, AOX (Ciona intestinalis, DM193474.1) open reading frame was cloned into the lentiviral vector (sequencing primers, Forward: 5′-CGCAAATGGGCGGTAGGCGTG-3′, Reverse: 5′-AGAGACAGC-AACCAGGAT-3′). AOX overexpression was achieved in Lovo cells with stable CKMT1 knockdown via lentivirus transfection. The expression of AOX was verified via qPCR (data not shown).

Immunohistochemistry (IHC)

IHC was performed as described previously[1]. Briefly, paraffin sections of colonic tissues were dewaxed and rehydrated. Then, sections underwent antigen retrieval, followed by blocking with 10% goat serum for 1h at room temperature. Tissues were incubated with CKMT1A antibody (1:200, Proteintech,15346-1-AP) overnight at 4 °C, and washed three times with PBS. The sections were incubated for 30 min at room

temperature with horseradish peroxidase-conjugated secondary antibody and visualized by substrate DAB (IHC Detect Kit, Proteintech, PK10006). Images were obtained by a brightfield microscopy (Olympus, Japan).

Immunofluorescence (IF)

Frozen sections of colonic tissues or cells (plated in confocal dishes) were used for IF staining. Briefly, samples were blocked with 10% goat serum for 1h at room temperature and then incubated with CKMT1A antibody (1:200, Proteintech,15346-1-AP), Occludin antibody (1 μg/mL, Invitrogen, 33-1500), EpCAM antibody (1:800, CST, 2929S), F4/80 (1:1000, CST, 70076), Ly-6G (1:1000, CST, 87048), CD86 (1:500, Proteintech, 13395-1-AP), or Tomm20 antibody (1:200, Santa Cruz, sc-17764) overnight at 4 °C. After washing 3 times with PBS, samples were incubated with Alexa Fluor 488- or Cy3-labeled secondary antibody (1:200, Servicebio, GB21303 or GB25303) for 1h at room temperature (protected from light). DAPI (Servicebio, G1012) was used for staining nuclear. Expression and localization of the proteins were analyzed with confocal fluorescence microscopy (MICA platform, Leica, Germany).

Proteomics sequencing (4D Label free)

Proteomics sequencing was performed with the assistance of PTM BIO Technologies (Hangzhou, China) CO. Colon tissues from KO^{IEC} and WT mice after DSS treatment were collected and analyzed by liquid chromatography-mass spectrometry (LC-MS). Briefly, tissues were grinded with liquid nitrogen and incubated with lysis buffer (8 M urea, 1% protease inhibitor cocktail), followed by sonication three times on ice. The supernatants of the lysates were obtained by centrifugation at 12,000 g at 4°C for 10 min. For trypsin digestion, dithiothreitol (5 mM, 30 min at 56°C) was used to reduce the protein solution. Iodoacetamide (11 mM, 15 min at room temperature) was used to alkylate in darkness. The sample was then diluted with 100 mM TEAB to urea concentration less than 2 M. Trypsin was used for final digestion. The peptides were desalted via C18 SPE column. The peptides were then dissolved in solvent A (0.1% formic acid, 2% acetonitrile/in water), and separated with a gradient solvent B (0.1%

formic acid in acetonitrile, 90% acetonitrile/in water), using the EASY-nLC 1200 system (Thermo). Database (Mus_musculus_10090_SP_20230103.fasta) was used for data analysis via Proteome Discoverer (v2.4.1.15).

TUNEL staining

To detect apoptosis *in situ*, paraffin sections of colonic tissues were used for TUNEL staining with TUNEL Cell Apoptosis Detection Kit (Servicebio, China), according to the manufacturer's instructions. Briefly, paraffin sections of colonic tissues were dewaxed and rehydrated. Proteinase K working liquid (20 μg/mL) was added to cover the sample and incubated for 20 min at 37°C. The section was gently washed with PBS for three times and incubated with 50 μL Equilibration Buffer for 10 min at 37°C. After removal of Equilibration Buffer, TdT incubation buffer, which was prepared on ice at the proportion of 2: 5: 50(Recombinant TdT enzyme: TMR-5-dUTP Labeling Mix: Equilibration Buffer), was added to the sample, and incubated for 60 min at 37°C in darkness. The sample was wash with PBS for 4 times and DAPI (Servicebio, China) was used for nuclear counterstaining before pictures were taken.

ELISA

The levels of interleukin (IL)-1β, tumor necrosis factor-α (TNF-α), IL-17, and IL-10 in the colon tissues were measured via ELISA with commercial ELISA kits (R&D) according to the manufacturer's instructions.

Transmission electron microscope (TEM)

Colon tissues (about 0.2cm × 0.2cm) were harvested quickly from mice and fixed in TEM fixative (2.5% glutaraldehyde in 0.1M PBS buffer, Servicebio, G1102) for 2h at room temperature (protected from light). Fixation process was continued overnight at 4 °C. The samples were then processed in a routine procedure with post-fix, dehydration, embedding, and sectioning. At last, samples were examined with a FEI TECNAI G2 20 TWIN transmission electron microscopy.

NADH/NAD$^+$ quantification

The NAD$^+$ and NADH concentrations were measured by a NAD$^+$/NADH Assay Kit via WST-8 (Beyotime, S0175) according to the manufacturer's instructions. Briefly,

approximate 1 × 10^6 cells were harvested and lysed with 200 μL NADH/NAD^+ extracting solution. Supernatant was collected after centrifugation at 12,000 g for 5min at 4 °C. Half of the supernatant (50-100μL) was heated at 60 °C for 30 min in water bath to decompose NAD^+, and then used for NADH detection. The rest of the supernatant was used for measuring the total level of NADH and NAD^+. Supernatant (20 μL) was transferred to the 96-well plate and incubated with working liquids of alcohol dehydrogenase for 10 min at 37°C. Developing solution was added and incubated for 30 min at 37°C. The absorbance (450 nm) was measured with SpectraMax Microplate reader (Molecular Devices, USA) for the analysis of the ratio of NADH/NAD^+.

Detection of apoptosis via flow cytometry

Cell apoptosis was detected with an Annexin V-FITC/propidium iodide (PI) apoptosis detection kit (Beyotime, C1062) according to the manufacturer's instructions. Briefly, approximate 1 × 10^5 cells were gently harvested by trypsin without EDTA and resuspended in Annexin V-FITC binding buffer. Annexin V-FITC and PI were added successively and mixed. After incubation for 10min at room temperature, cells were kept on an ice bath and immediately detected by flow cytometry.

Mitochondrial permeability transition pore (mPTP) measurement

The opening of mPTP was analyzed with a mPTP assay kit (Beyotime, C2009S) according to the manufacturer's protocol. Briefly, approximate 1 × 10^6 cells were harvested and resuspended in 1 mL staining solution (Calcein AM plus $CoCl_2$ quencher) at 37°C for 30 min (protected from light). For ionomycin (a calcium ionophore) control, ionomycin (500 nM, final concentration) was added into staining solution to stimulate mPTP opening. After incubation, cells were gently washed and resuspended with a detection buffer. Finally, the fluorescence was immediately detected by flow cytometry.

Mitochondrial morphology analysis

Mitochondria in living cells (NCM460) was observed via super-resolution confocal imaging. Briefly, cells were washed with prewarmed HBSS twice and then incubated with PK Mito Orange Dye[2] (Genvivo Biotech, diluted in cell medium, 300 nM) in

cell incubator for 10 min. Upon completion of incubation, cells were washed gently with prewarmed cell medium twice and observed immediately under a confocal fluorescent microscope (Zeiss LSM900 with Airyscan). The mitochondria network was analyzed with Image J software and MiNA plugin[3].

Mitochondria isolation

Mitochondria were extracted using a Mitochondria Isolation Kit for Cultured Cells (Thermo Scientific, 89874) according to the manufacturer's manual. Briefly, about 2 $\times 10^7$ cells were harvested in a 2.0 mL microcentrifuge tube after centrifugation at 850 g for 2 min. The tube was added with 800 μL Mitochondria Isolation Reagent A, vortexed for 5 sec and incubated on ice for 2 min. The sample was then added with Mitochondria Isolation Reagent B (10 μL) and vortexed for 5 sec at maximum speed. The sample was incubated on ice for 5 minutes with vortexing at maximum speed every 60 sec. Mitochondria Isolation Reagent C (800 μL) was added and mixed via inverting the tube for 5 times. The supernatant was obtained after centrifugation at 700 g for 10 min at 4 °C and transferred to a new 2 mL tube. The tube was then centrifugated at 3000 g for 15 minutes at 4°C. Cytosol fraction (the supernatant) was obtained. The mitochondria fraction in the pellet was washed again with Mitochondria Isolation Reagent C (500 μL) and finally obtained after centrifugation at 12000 g for 5 min.

References

[1] Wang SL, Shao BZ, Zhao SB, Chang X, Wang P, Miao CY, et al. Intestinal autophagy links psychosocial stress with gut microbiota to promote inflammatory bowel disease. Cell Death Dis. 2019. 10(6): 391.

[2] Yang Z, Li L, Ling J, Liu T, Huang X, Ying Y, et al. Cyclooctatetraene-conjugated cyanine mitochondrial probes minimize phototoxicity in fluorescence and nanoscopic imaging. Chem Sci. 2020. 11(32): 8506-8516.

[3] Valente AJ, Maddalena LA, Robb EL, Moradi F, Stuart JA. A simple ImageJ macro tool for analyzing mitochondrial network morphology in mammalian cell culture. Acta Histochem. 2017. 119(3): 315-326.

Supplementary Tables

Table 1. Antibodies for Western blot

Antibody	Source	Company	Product NO.	Dilution
Anti-CKMT1A Antibody	Rabbit	Proteintech	15346-1-AP	1:1000 (WB)
Anti-Bax Antibody	Rabbit	Proteintech	50599-2-Ig	1:5000 (WB)
Anti-GAPDH Antibody	Mouse	Engibody	AT0002	1:5000 (WB)
Anti-β-Actin Antibody (HRP Conjugated)	--	Servicebio	ZB15001-HRP	1:2000 (WB)
Anti-PARP1 Antibody	Rabbit	Proteintech	13371-AP	1:2000 (WB)
Anti-Cytochrome c Antibody	Rabbit	Proteintech	10993-1-AP	1:2000 (WB)
Anti-Claudin 1 Antibody	Rabbit	AiFang Bio	AF301298	1:2000 (WB)
Anti-VDAC1 Antibody	Mouse	Santa Cruz	sc-390996	1:200 (WB)
Anti-E-Cadherin Antibody	Rabbit	CST	3195T	1:1000 (WB)
Anti-AIF Antibody	Mouse	Santa Cruz	sc-13116	1:500 (WB)
Anti-Caspase-3 Antibody	Rabbit	CST	14220	1:1000 (WB)
Anti-C-Caspase-3 Antibody	Rabbit	CST	9664	1:1000 (WB)
Anti-Occludin Antibody	Rabbit	Abclonal	A2601	1:1000 (WB)
Anti-α-Tubulin Antibody	Mouse	GenScript	A01410	1:5000 (WB)
Anti-SOD2 Antibody	Rabbit	Abclonal	A19576	1:1000 (WB)
Anti-FIS1 Antibody	Rabbit	Proteintech	10956-1-AP	1:5000 (WB)
Anti-PRDX5 Antibody	Rabbit	Abclonal	A24731	1:2000 (WB)
Anti-GPX4 Antibody	Rabbit	Abclonal	A21440	1:1000 (WB)
Anti-TXNRD2 Antibody	Rabbit	Abclonal	A8884	1:1000 (WB)
Anti-PRDX3 Antibody	Rabbit	Proteintech	10664-1-AP	1:5000 (WB)
Anti-GPX1 Antibody	Rabbit	Proteintech	29329-1-AP	1:5000 (WB)
Anti-NE Antibody	Mouse	Santa Cruz	sc-55549	1:200 (WB)
Anti-Bcl-2 Antibody	Rabbit	Proteintech	12789-1-AP	1:1000 (WB)
Anti-MFN1 Antibody	Rabbit	Proteintech	13798-1-AP	1:5000 (WB)
Anti-PGC-1α Antibody	Mouse	Santa Cruz	sc-517380	1:500 (WB)
Anti-ATP5A Antibody	Mouse	Santa Cruz	sc-136178	1:500 (WB)
Anti-FIS1 Antibody	Rabbit	Proteintech	10956-1-AP	1:5000 (WB)
Anti-SPG7 Antibody	Rabbit	Proteintech	27801-1-AP	1:1000 (WB)
Anti-MPO Antibody	Rabbit	Abcam	ab208670	1:1000 (WB)

Table 2. qPCR primer list

Name	Sequences
Mouse *Prdx3* Forward	5' GACTTCTTGATGGCTAACCAAT-3'
Mouse *Prdx3* Reverse	5' GAATGCTTACCCTCTAAGGACA-3'
Mouse *Tomm20* Forward	5' AGC AGC ATT CTC ACA GGA CAG 3'
Mouse *Tomm20* Reverse	5' TCT TCC CAC TAC ACT GAC CCA 3'
Mouse *Txnrd2* Forward	5' AAT AGA AGG CTC CTC AGA CAC C 3'
Mouse *Txnrd2* Reverse	5' ACA CGA TGT TCC ATC CAC AT 3'
Mouse *Atp5a1* Forward	5' CAG TTT GGT TCT GAT TTG GAC 3'
Mouse *Atp5a1* Reverse	5' CGT ACA CCC GCA TAG ATA ACA 3'
Mouse *Pparg* Forward	5' GGC AAA GCA TTT GTA TGA CTC 3
Mouse *Pparg* Reverse	5' GTG ATT TGT CCG TTG TCT TTC 3'
Mouse *Nrf1* Forward	5' TAC TCT GCT GTG GCT GAT GGA 3'
Mouse *Nrf1* Reverse	5' ATG CTT GCG TCG TCT GGA T 3'
Mouse *Ppargc1a* Forward	5' ATC TGG GTG GGA GAG GAT ACT 3'
Mouse *Ppargc1a* Reverse	5' TAG GTG TCA GGA CAA AGG ACA 3'
Mouse *Tfam* Forward	5' CGG AGA CAT CTC TGA GCA TTA 3'
Mouse *Tfam* Reverse	5' AAG GCT TTG AGA CCT AAC TGG 3'
Mouse *Nfe2l2* Forward	5' ATG GAG CAA GTT TGG CAG G 3'
Mouse *Nfe2l2* Reverse	5' TGG GAA CAG CGG TAG TAT CAG 3'
Mouse *Prdx5* Forward	5' TGG TGT CTC TCT TTG GGA ATC 3'
Mouse *Prdx5* Reverse	5' GCC TTC ACT ATG CCG TTG T 3'
Mouse *Gpx1* Forward	5' CGA ACC TGA CAT AGA AAC CCT 3'
Mouse *Gpx1* Reverse	5' AGC CAG TAA TCA CCA AGC C 3'
Mouse *Sod2* Forward	5' CAA GGT GAC ACA GTG CTT CTG 3'
Mouse *Sod2* Reverse	5' GGA TGA CAG GAA GAT GGT GAG 3'
Mouse *Ppara* Forward	5' GAG GAT GGG GAC TTT TGT TCT 3'
Mouse *Ppara* Reverse	5' GGC TTT TTG GCT GTA GGA GG 3'
Mouse *Gpx4* Forward	5' TGT GGA AAT GGA TGA AAG TCC 3'
Mouse *Gpx4* Reverse	5' GCA GCC GTT CTT ATC AAT GAG 3'
Mouse *Tbp* Forward	5' ACCGTGAATCTTGGCTGTAAAC 3'
Mouse *Tbp* Reverse	5' GCAGCAAATCGCTTGGGATTA 3'
Mouse *Opa1* Forward	5' GTG GAT GTA CTG GAA GAA TCG 3'
Mouse *Opa1* Reverse	5' GTG GTA ATC TCA TCA CTT GCC 3'

Mouse *Dnm1l* Forward	5' GGA TCA TTC AGC ATT GTA GCA 3'
Mouse *Dnm1l* Reverse	5' GGC AAC CTT TTA CGA AGA AGA 3'
Mouse *Mfn2* Forward	5' CAA GAA GGA TAA GCG ACA CAT 3'
Mouse *Mfn2* Reverse	5' CTG CTC AAA GAT TCC ATT GAT 3'
Mouse *Fis1* Forward	5' TAA AGT ATG TGC GAG GGC TGT 3'
Mouse *Fis1* Reverse	5' CTT ATC AAT CAG GCG TTC CAG 3'
Mouse *Mfn1* Forward	5' AAG CAG TTT CTA CAC CCG AGC 3'
Mouse *Mfn1* Reverse	5' CAT TTC CCA TCT TCC AAG TCC 3'
Mouse *Pink1* Forward	5' CCC GTC CAG TTA GGT TCT TG 3'
Mouse *Pink1* Reverse	5' CCT TTG CCT CAG TGA TAG GTT AG 3'
Mouse *Tnf* Forward	5' CCCCAAAGGGATGAGAAGT3'
Mouse *Tnf* Reverse	5' GTTTGTGAGTGTGAGGGTCTG3'
Mouse *Il17a* Forward	5' GTT CGT GCT ATT GAT TTT CAG C 3'
Mouse *Il17a* Reverse	5' GGA CCC CTT TAC ACC TTC TTT 3'
Mouse *Il1b* Forward	5' TAC ATC AGC ACC TCA CAA GC 3'
Mouse *Il1b* Reverse	5' AGA AAC AGT CCA GCC CAT ACT 3'
Human GAPDH Forward	5' CCTCTGACTTCAACAGCGACA 3'
Human GAPDH Reverse	5' ATGAGCTTGACAAAGTGGTCGT 3'
Human CKMT1A Forward	5' AGAGGTGGAACGTGTTGTGG 3'
Human CKMT1A Reverse	5' CAACGGGGACACAGGCTTAT 3'

Table 3. Reagents used in the study

**Reagents**	**Company**	**Product NO.**	**Concentration used**
Recombinant Human TNF-α	PeproTech	300-01A	50 ng/ml or 200 ng/mL
Recombinant Murine TNF-α	PeproTech	315-01A	50 ng/mL
IFN-gamma, Mouse	MedChemExpress	HY-P7071	50 ng/mL
Rotenone	MedChemExpress	HY-B1756	10 nM (pretreat for 3h)
Diazoxide	MedChemExpress	HY-B1140	200 nM (pretreat for 3h)
Cycloheximide	MedChemExpress	HY-12320	20 μg/mL
FCCP	MedChemExpress	HY-100410	100 nM (pretreat for 3h)

**Reagents**	**Company**	**Product NO.**	**Concentration used**
Cyclosporin A	MedChemExpress	HY-B0579	1 μM (pretreat for 3h)
Staurosporine	MedChemExpress	HY-15141	2 μM
N-acetylcysteine (NAC)	Sigma	A7250	5mM (pretreat for 3h)
