# Supplementary material for: CKMT1 deficiency contributes to mitochondrial dysfunction and promotes intestinal epithelial cell apoptosis via reverse electron transfer-derived ROS in colitis
Source: Cell Death Dis. 2025 Mar 15;16(1):177. doi: 10.1038/s41419-025-07504-4 (PMC11910573; doi:10.1038/s41419-025-07504-4)

Figure 1
Unprocessed original images of western blots

CKMT1

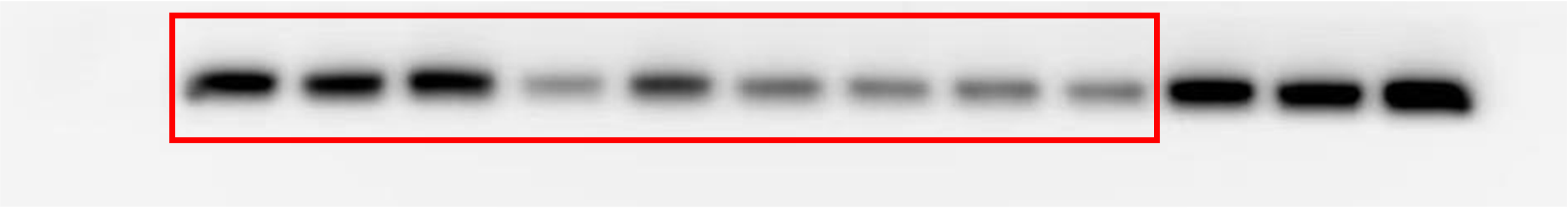

GAPDH

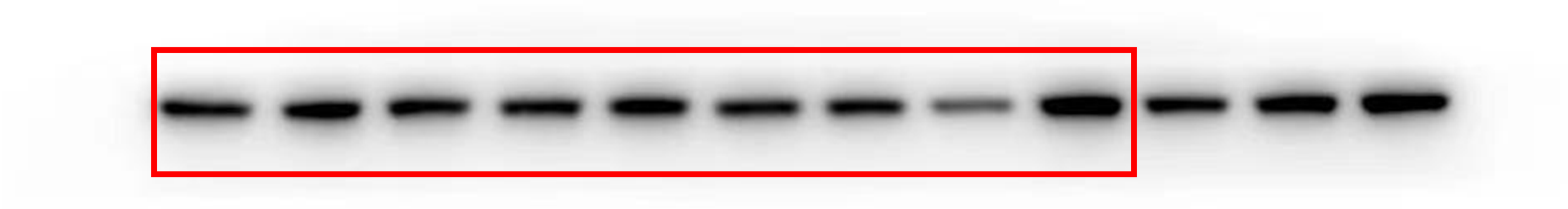

CKMT1

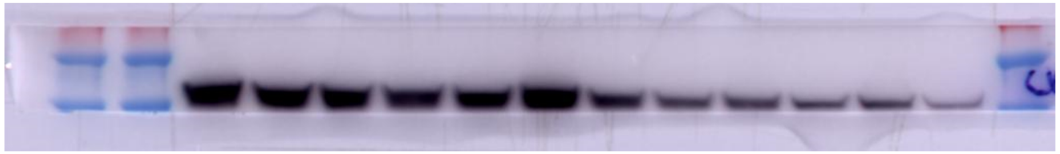

GAPDH

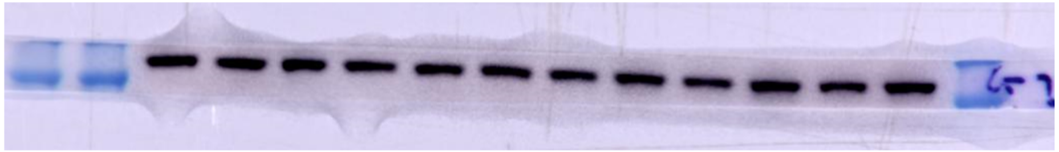

Figure 2
Unprocessed original images of western blots

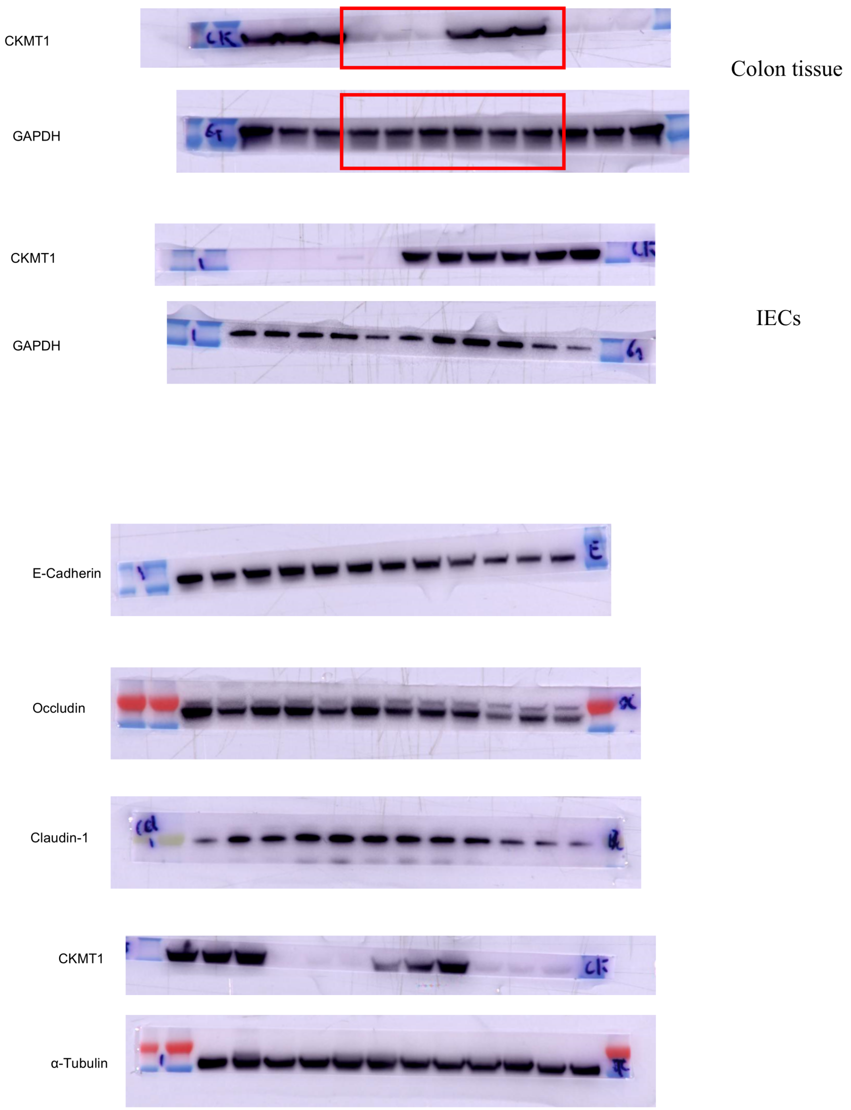

Figure 3
Unprocessed original images of western blots

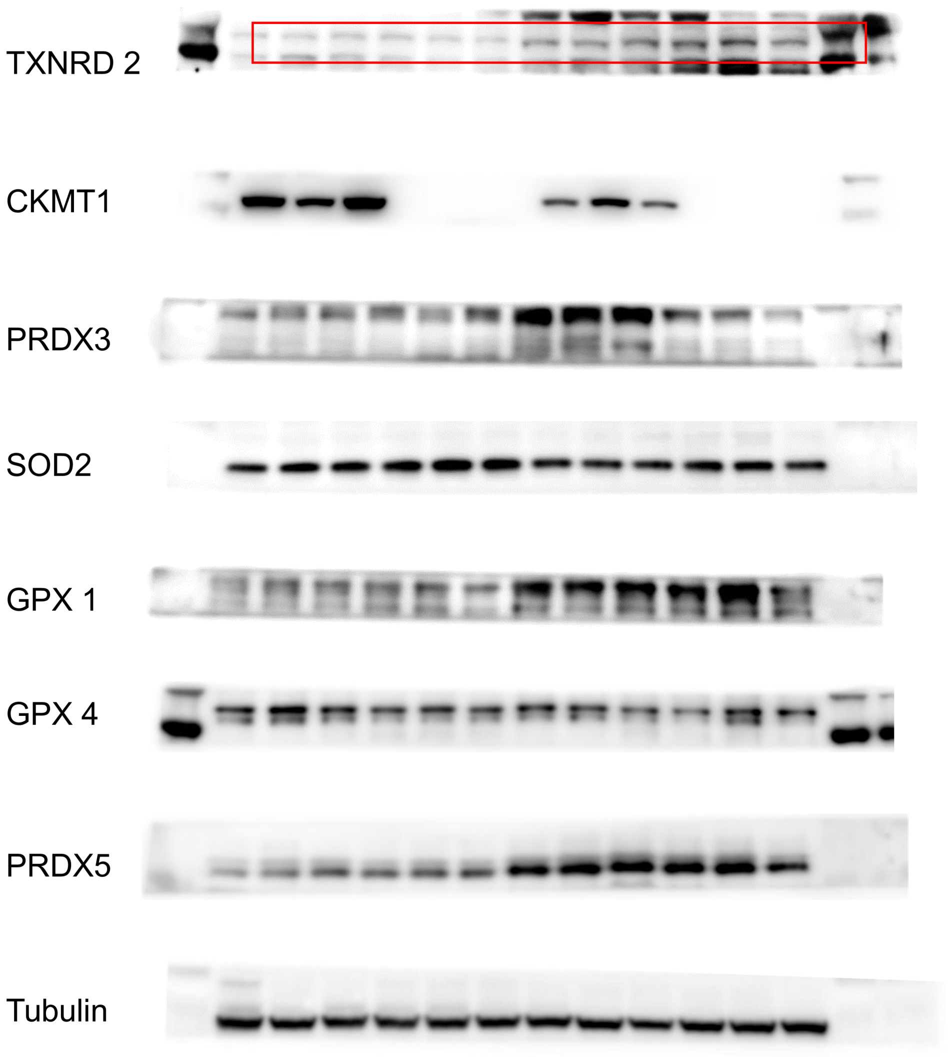

Figure 4

Unprocessed original images of western blots

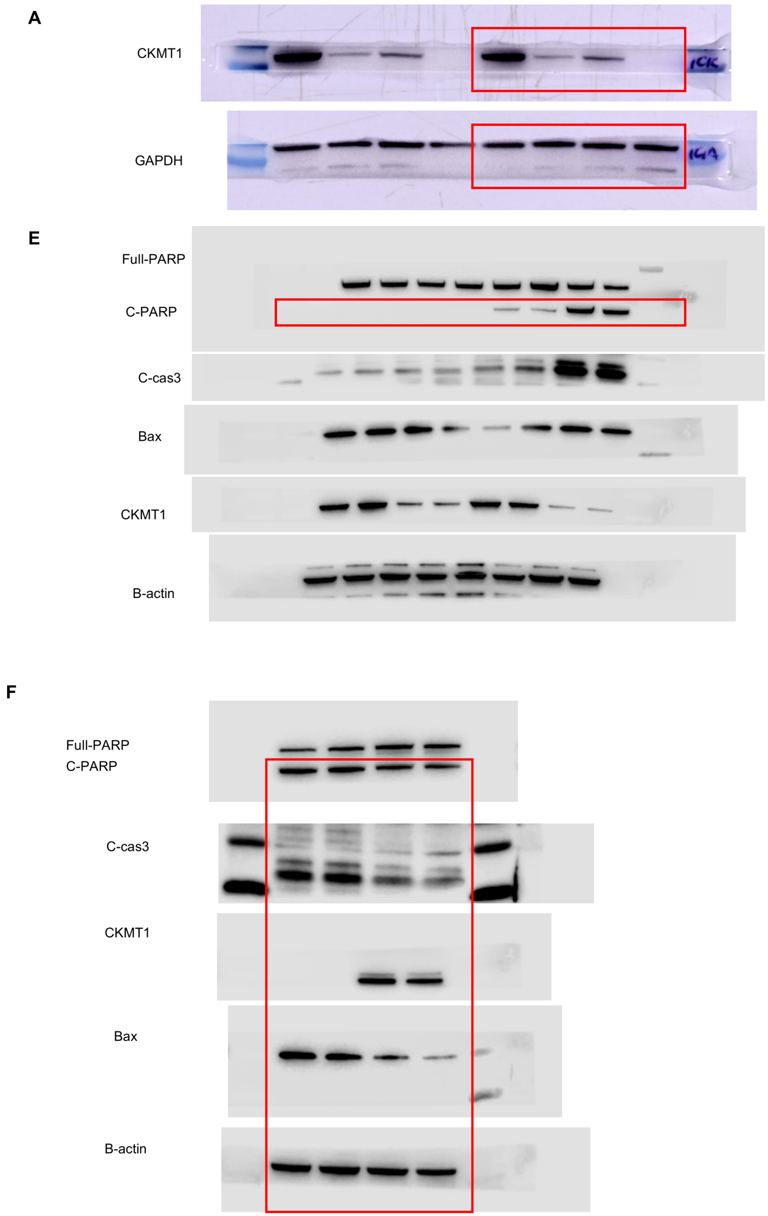

Figure 4
Unprocessed original images of western blots

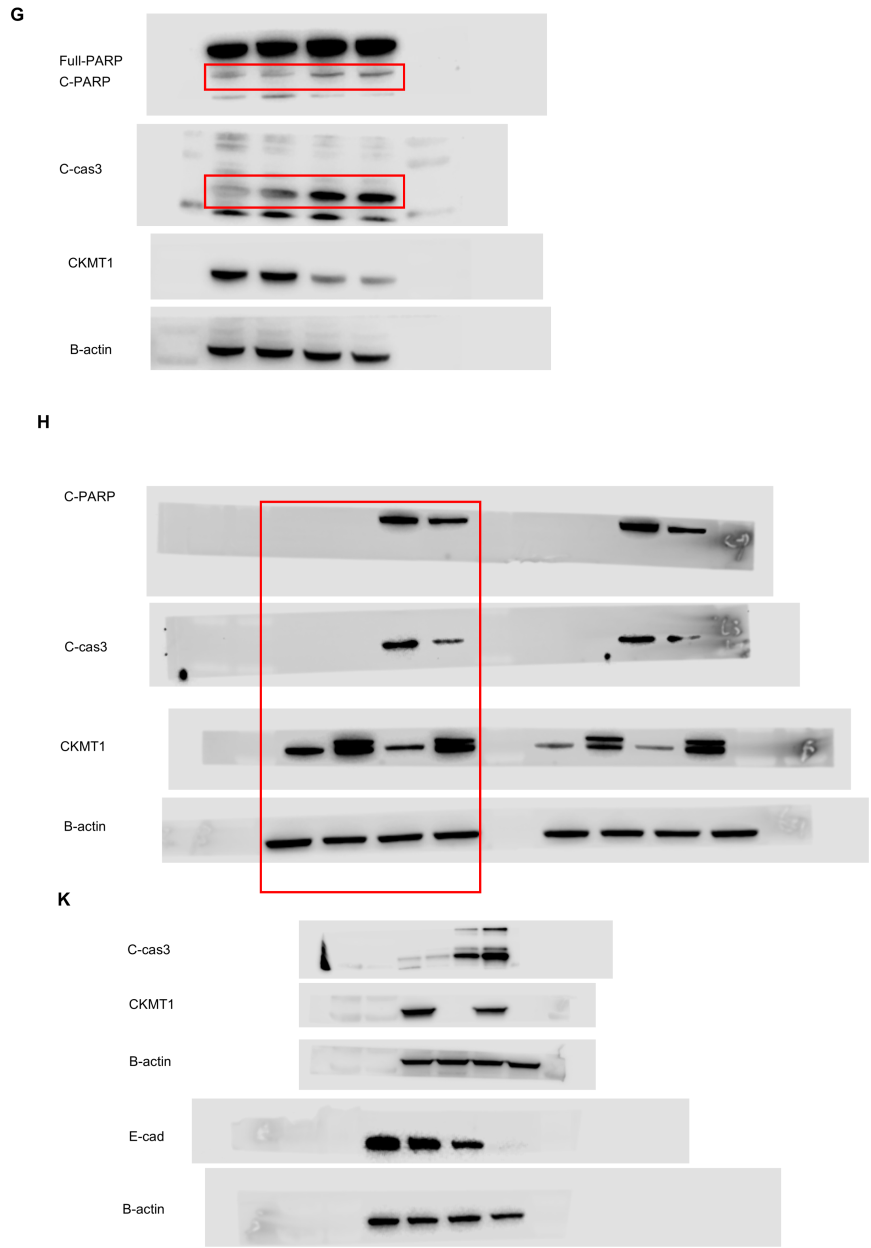

Figure 6
Unprocessed original images of western blots

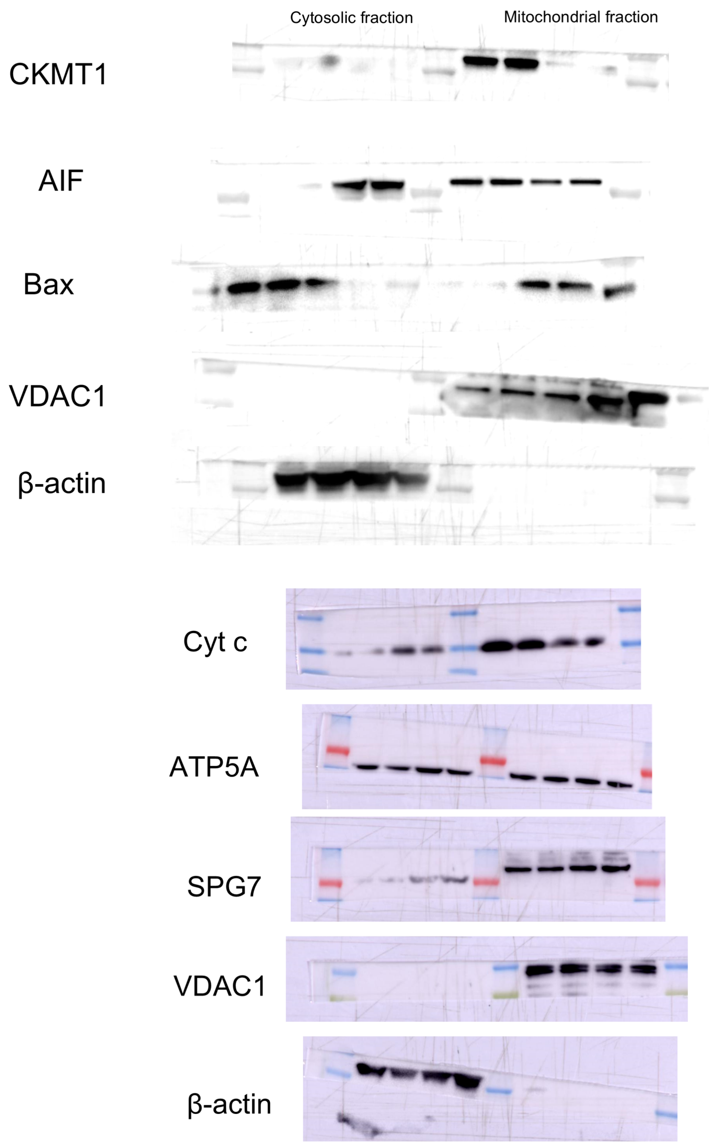

Figure 6
Unprocessed original images of western blots

F

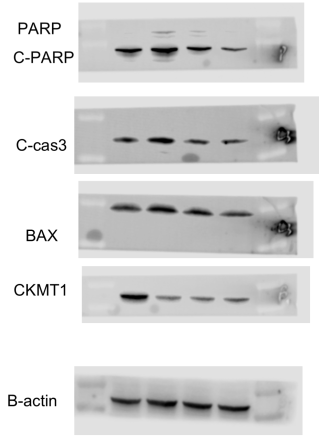

Figure S3
Unprocessed original images of western blots

CKMT1

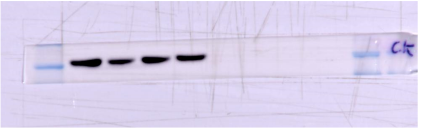

MPO

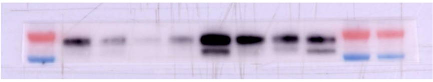

NE

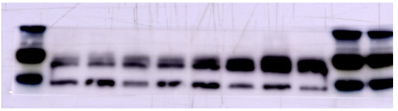

α-Tubulin

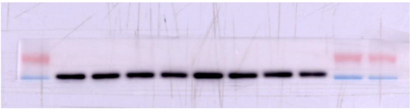

Figure S4

Unprocessed original images of western blots

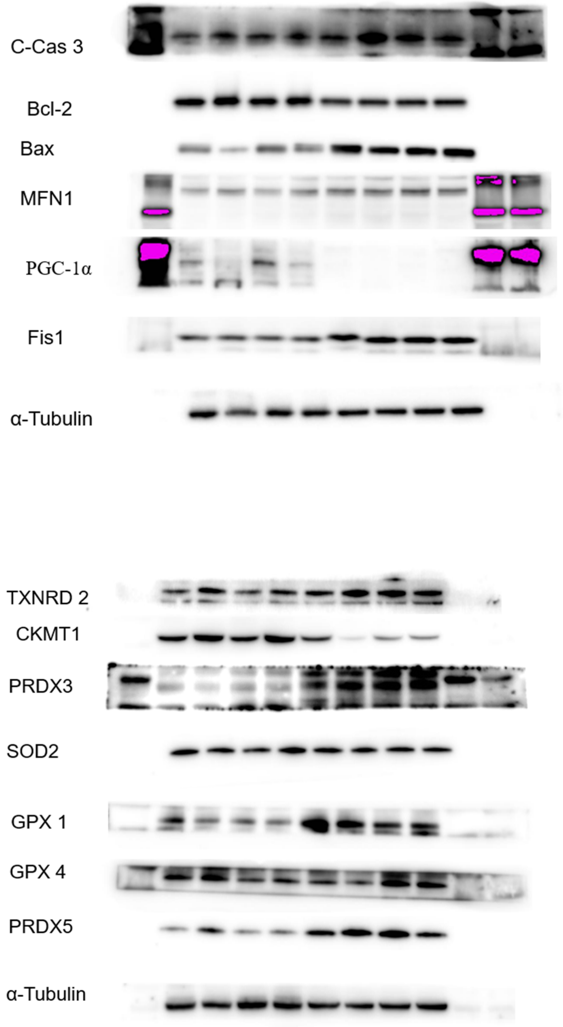

Figure. S5
Unprocessed original images of western blots

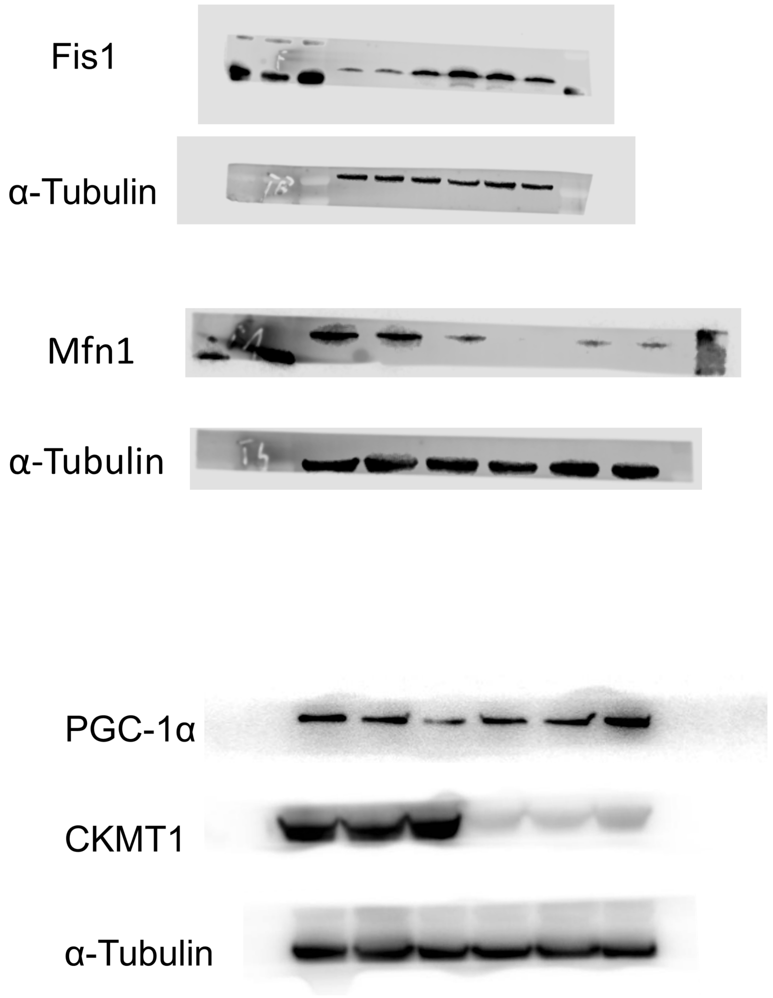

Figure. S6

Unprocessed original images of western blots

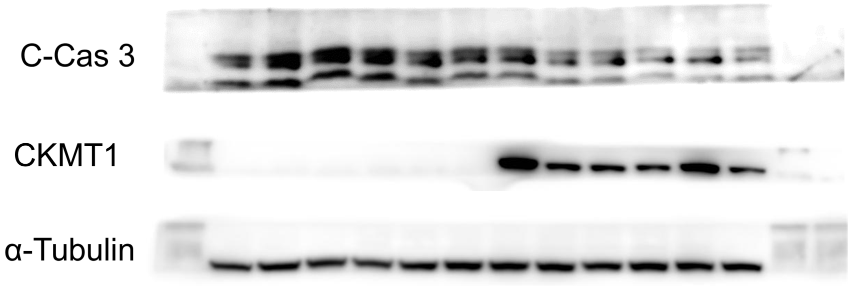

Supplement: Supplementary file 2 — Original Data for Western Blotting [file 41419_2025_7504_MOESM2_ESM.pdf]
